# Structural and Functional Basis of GenB2 Isomerase Activity from Gentamicin Biosynthesis

**DOI:** 10.1021/acschembio.4c00334

**Published:** 2024-08-29

**Authors:** Gabriel S. de Oliveira, Priscila dos S. Bury, Fanglu Huang, Yuan Li, Natália C. de Araújo, Jiahai Zhou, Yuhui Sun, Finian J. Leeper, Peter F. Leadlay, Marcio V. B. Dias

**Affiliations:** † Department of Microbiology, Institute of Biomedical Sciences, University of Sao Paulo, Sao Paulo 05508-000, Brazil; ‡ Department of Biochemistry, University of Cambridge, Cambridge CB2 1GA, U.K.; § Key Laboratory of Combinatorial Biosynthesis and Drug Discovery (Ministry of Education), and School of Pharmaceutical Sciences, Wuhan University, Wuhan 430071, China; ∥ State Key Laboratory of Quantitative Synthetic Biology, Shenzhen Institute of Synthetic Biology, Shenzhen Institute of Advanced Technology, CAS, Shenzhen 518055, China; ⊥ Yusuf Hamied Department of Chemistry, University of Cambridge, Cambridge CB2 1EW, U.K.

## Abstract

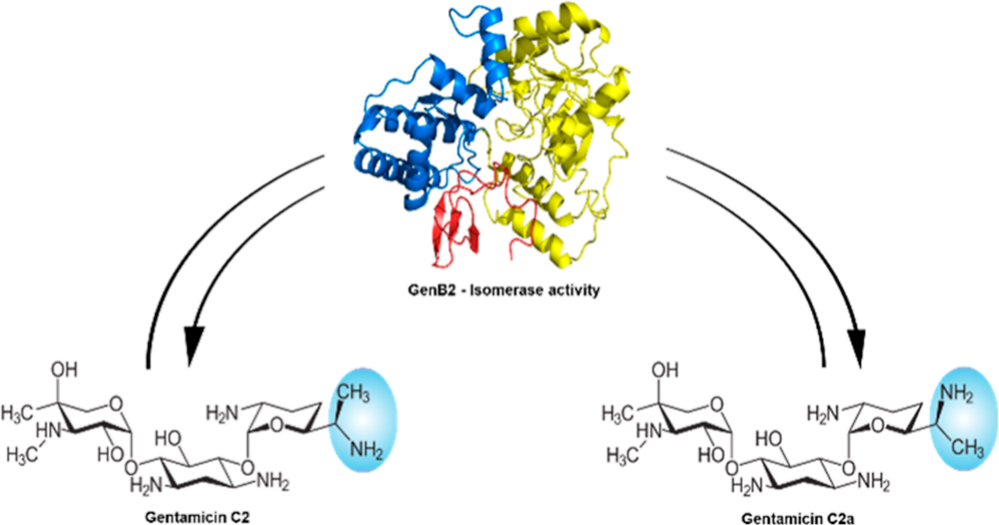

Aminoglycosides are essential antibiotics used to treat severe infections caused mainly by Gram-negative bacteria. Gentamicin is an aminoglycoside and, despite its toxicity, is clinically used to treat several pulmonary and urinary infections. The commercial form of gentamicin is a mixture of five compounds with minor differences in the methylation of one of their aminosugars. In the case of two compounds, gentamicin C2 and C2a, the only difference is the stereochemistry of the methyl group attached to C-6′. GenB2 is the enzyme responsible for this epimerization and is one of the four PLP-dependent enzymes encoded by the gentamicin biosynthetic gene cluster. Herein, we have determined the structure of GenB2 in its holo form in complex with PMP and also in the ternary complex with gentamicin X2 and G418, two substrate analogues. Based on the structural analysis, we were able to identify the structural basis for the catalytic mechanism of this enzyme, which was also studied by site-directed mutagenesis. Unprecedently, GenB2 is a PLP-dependent enzyme from fold I, which is able to catalyze an epimerization but with a mechanism distinct from that of fold III PLP-dependent epimerases using a cysteine residue near the N-terminus. The substitution of this cysteine residue for serine or alanine completely abolished the epimerase function of the enzyme, confirming its involvement. This study not only contributes to the understanding of the enzymology of gentamicin biosynthesis but also provides valuable details for exploring the enzymatic production of new aminoglycoside derivatives.

## Introduction

**Figure 1:**
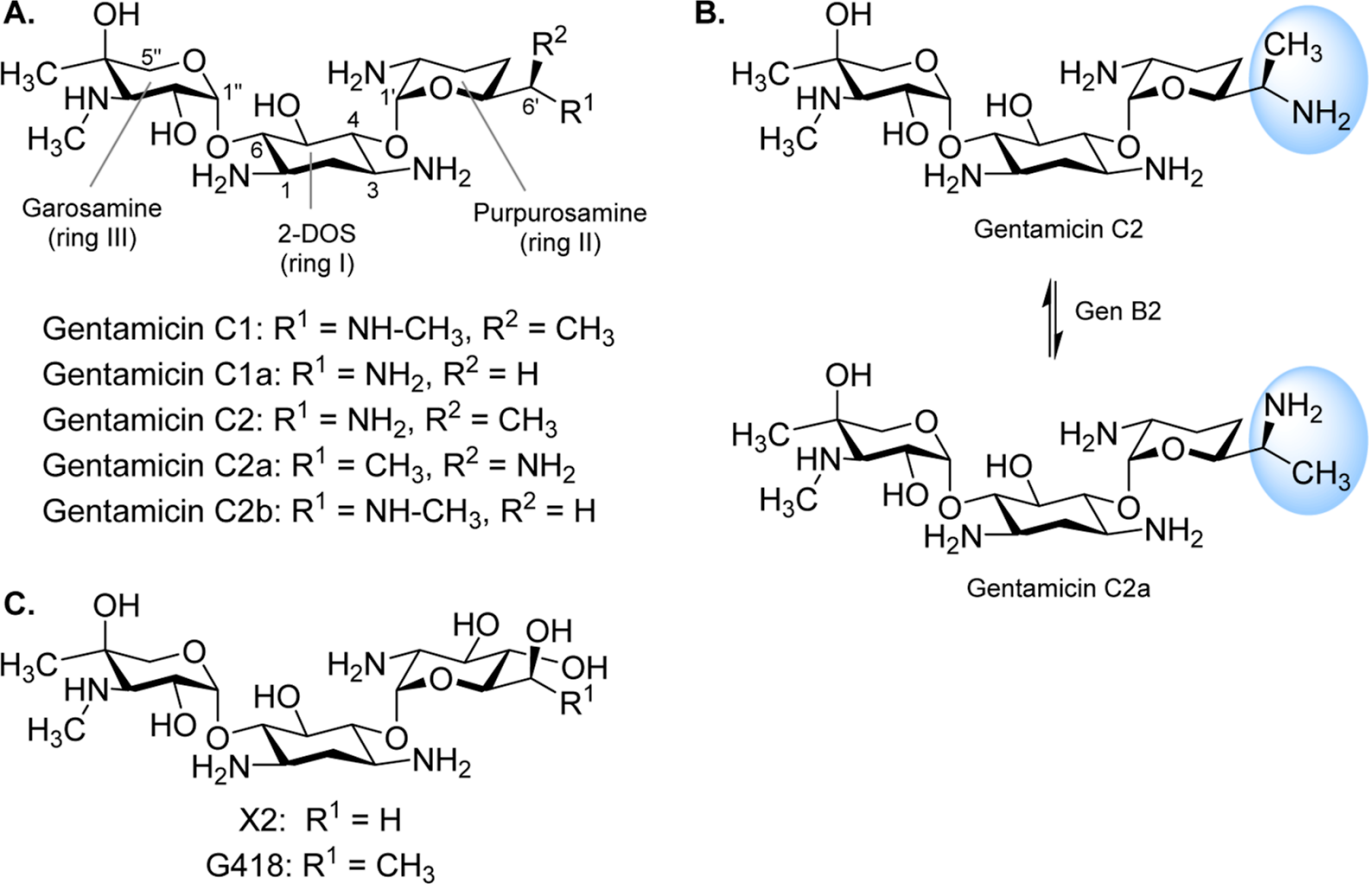
Gentamicin C complex. (A) Chemical structure of individual components of the gentamicin C complex (2-DOS means 2-deoxystreptamine). (B) Epimerase reaction catalyzed by GenB2. In blue is shown the group that undergoes epimerization. (C) Chemical structures of the two gentamicin precursors used in this study.

Aminoglycosides are an important class of antibiotics used to treat several bacterial infections, particularly those caused by Gram-negative strains.^1−3^ These antibiotics are highly functionalized molecules derived from the glycolytic pathway and usually contain an aminocyclitol, including 2-deoxystreptamine (2-DOS), as an aglycone.^4,5^ These molecules inhibit protein synthesis by binding to the A site in the 30S subunit of bacterial ribosomes and interfering with the molecular basis of translation fidelity.^6^ Gentamicin is one of the most functionalized aminoglycosides. The commercial form is a mixture containing five components, which have differences in the methylation level and stereochemistry of a methyl group at position C-6′ of their unusual sugar rings.^2^ These components are denominated as gentamicin complex C (C1, C1a, C2, C2a, and C2b) (Figure 1),^7^ and due to their minor differences, the separation of each component is challenging.^8^ The individual components differ in their nephrotoxic and ototoxic effects,^9^ so the particular properties of each compound are a strategy to be explored for safer use or in the development of new gentamicin derivatives.^7^ Gentamicin is primarily used in lung infections, particularly in patients with cystic fibrosis and urinary infections, which are difficult to treat,^10,11^ but its use is restricted due to its toxic effects.^12,13^

The biosynthetic gene cluster (BGC) to produce gentamicins has been identified and annotated in *Micromonospora echinospora*,^14^ and it is highly similar to sisomicin produced by *Micromonospora inyoensis*.^15^ These BGCs include a number of exclusive genes not found in any other aminoglycoside BGCs,^5^ which are responsible for the methylations and deoxygenations found in these aminoglycosides. Gentamicins and sisomicin, in addition to the 2-DOS (ring I) moiety, both have two other hexoses, purpurosamine in gentamicin C1a or its dehydro-derivative sisosamine in sisomicin (ring II) and garosamine (ring III) linked to positions 4 and 6 of 2-DOS.^14^ Since the discovery of gentamicin and sisomicin BGCs,^14,15^ great progress has been achieved in understanding the biosynthesis of these two aminoglycosides.^16^ In recent years, we and others have focused on elucidating the enzymes involved in producing C components of gentamicin, including their 3D-structure determination and functional and biochemical validation.^7,17−25^ The biosynthesis of gentamicin, similarly to other aminoglycosides, starts with modifying glucose 6-phosphate by a series of enzymes to produce 2-DOS. Two glycosyltransferases, GenM1 and GenM2, are responsible for attaching N-acetylglucosamine, which is deacetylated, and xylose, respectively, to 2-DOS to produce gentamicin A.^25^ From gentamicin A2, a series of functionalizations by specific enzymes from gentamicin or sisomicin BGCs occur to produce the precursor gentamicin X2 and generate products from the gentamicin C complex. GenK has a pivotal role in this process by methylating C-6′ of gentamicin X2, producing G418, and branching the biosynthesis of gentamicin into two pathways, in which gentamicin X2 and G418 each undergo a series of modifications.^7,19,26^ Four genes encoding PLP-dependent enzymes (GenB1–GenB4) identified in the gentamicin BGC and a methyltransferase, GenL, from outside the BGC, are responsible for the last steps of gentamicin C complex biosynthesis.^7,20^ GenB1 was confirmed to be the major transaminase, which, together with dehydrogenase GenQ, converts the alcohol at C-6′ of X2 and G418 to an amine to produce the branched intermediates JI-20A and JI-20-Ba, respectively.^20^ GenB3 and GenB4, together with kinase GenP, were recently confirmed by us and others to be involved in an unusual dideoxygenation of both intermediates to produce C1a and C2a, respectively.^21,22^ GenB2 acts as an isomerase, converting C2a into C2 by changing the stereochemistry of the chiral center C-6′ (Figure 1B).^20,24^ Finally, the last step of gentamicin biosynthesis involves GenL, which methylates the 6′-N of C1a and C2, producing C2b and C1, respectively.^7^

Although the 3D structures of three of the PLP-dependent enzymes (GenB1, GenB3, and GenB4) involved in the last steps of gentamicin complex C biosynthesis have been determined, and their molecular basis of catalysis has been proposed,^21,23,25^ the structure and molecular basis of the epimerase activity of GenB2 remain elusive.

PLP-dependent enzymes are very versatile proteins and catalyze a large number of different reactions, including transamination, decarboxylation, retro-aldol eliminations, and racemization, among others.^27^ Most PLP-dependent enzymes share a common mechanism involving the formation of an internal aldimine in which the cofactor PLP is covalently bonded to an active site lysine *via* a Schiff-base linkage (resting state). Upon substrate binding, a transaldimination reaction occurs, with the ε-amino group of the catalytic lysine being displaced by the substrate, producing an external aldimine. Also, in most cases, PLP plays a role as an electron sink, stabilizing a carbanion at the α-position and leading to the formation of the quinonoid intermediate.^27^ However, in recent years, the known mechanisms of PLP-dependent enzymes have become even more diverse,^28^ and PLP-dependent enzymes involved in the biosynthesis of natural products are reported to perform a vast range of reactions, often with surprising mechanisms.^27^

In the case of PLP-dependent enzymes that play a role as racemases or epimerases, the most studied family is the alanine racemases that catalyze the conversion of l-alanine to d-alanine in bacteria, which is a key compound of the cell wall in bacteria.^29^ Usually, these enzymes have a fold from class III of PLP-dependent enzymes, in which each protomer of the functional dimer has two domains: the N-terminal domain, constituted by an eight-stranded α/β-barrel, and the C-terminal domain, constituted mainly by a β-sheet.^30,31^ However, based on the sequence, GenB2 does not have similarity with class III PLP-dependent enzymes but has the class I fold, which also includes ornithine aminotransferases^32^ and the other PLP-dependent enzymes (GenB1, B3, and B4) identified in the gene cluster of gentamicin biosynthesis.^21^ This suggests that GenB2 should have an unprecedented mechanism of epimerase activity inside the type I PLP-dependent enzyme fold family.

In order to understand the structure and the epimerase mechanism of GenB2 in the biosynthesis of gentamicin, we have determined its 3-dimensional structure in its holo form and in complex with two alternative substrate analogues, G418 and gentamicin X2. The structure of GenB2 shows, as expected, a characteristic PLP-dependent enzyme class I fold. By analysis of the active site, it is possible to gain insight into the mechanism of the epimerase activity, and we propose a catalytic mechanism that involves an unprecedented cysteine residue from the N-terminal domain.

## Results and Discussion

GenB2 was successfully produced, and the enzyme purified close to homogeneity showed a pink color, indicating copurification with the coenzyme.^33^ This protein was submitted to several crystallization conditions, and we were able to produce large, thick, square-shaped crystals. Crystals of holo GenB2 and in complex with G418 and gentamicin X2 diffracted up to 1.35 Å, belonging to the space group *C*222_1_ and having a single protomer in the asymmetric unit. The active dimer of the enzyme can be obtained by using a symmetry operation and is the predominant form in solution. Although we have made several attempts to obtain the GenB2 structure in the resting complex (internal aldimine), we did not observe the formation of the Schiff base between the coenzyme and Lys227 in any of the tested crystal and solved structures. Based on that, we assumed that pyridoxamine 5-phosphate (PMP) is the predominant coenzyme state bound to the enzyme. The data processing, structure determination, and stereochemistry statistics can be observed in Table S1.

Performing a search on the DALI server, the returned structures, as expected, were those similar to ornithine aminotransferase, including NeoB, a transaminase involved in neomycin biosynthesis (which plays a similar role to that of GenB1)^23^ and GenB3, also from gentamicin biosynthesis.^21^ These structures have sequence similarity values of about 37 and 28% and a Z-score of 51.2 and 43.1, respectively. This indicates that GenB2 also has a type I PLP-dependent enzyme fold but with an epimerase function, which has so far not been described for this group of PLP-dependent enzymes. When the amino acid sequence of GenB2 is aligned with GenB1, GenB3, GenB4, and NeoB, we can observe that the most crucial residues for anchoring PLP and aminotransferase activity are conserved, which agrees with the residual aminotransferase activity of this enzyme^20^ (Figure S1).

**Figure 2:**
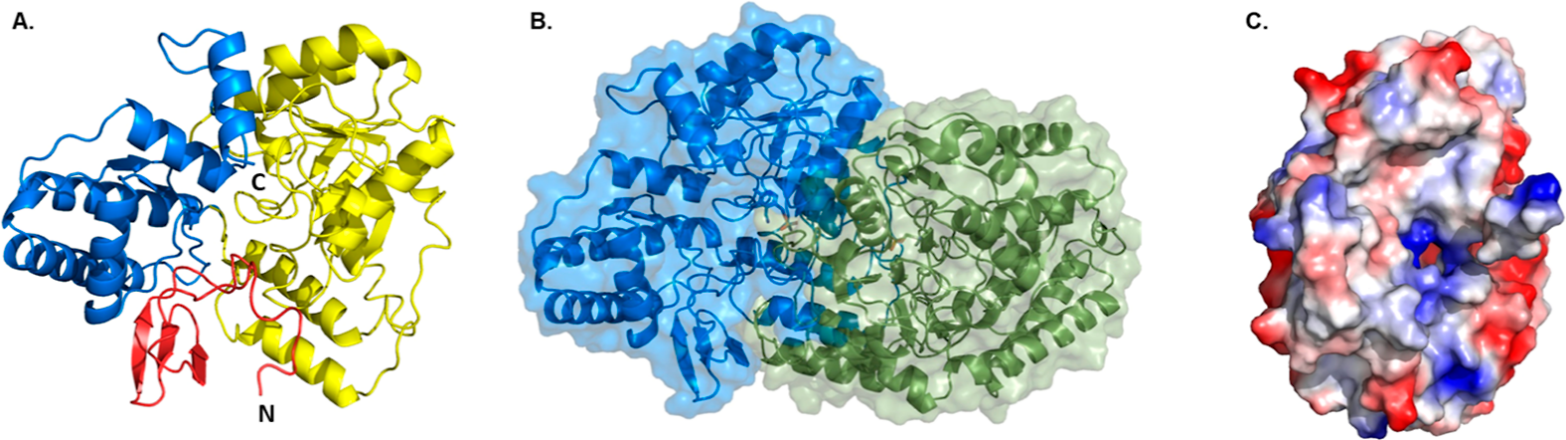
Overall structure of GenB2. (A) Representation of a protomer structure of GenB2. In red is the N-terminal domain, in yellow is the PLP binding domain, and in blue is the C-terminal domain. (B) Dimeric structure of GenB2 shows the organization of the two protomers (one in blue and the other in green) that form the dimeric structure of GenB2. (C) Electrostatic surface in the dimerization interaction of GenB2.

Similar to other members of the type I PLP-dependent enzyme fold family such as GenB1, GenB3, and GenB4, each protomer of GenB2 can be described to have three domains: a large domain that holds a 7-stranded mixed β-sheet with four parallel and three antiparallel strands and six α-helices (since PLP binds to this domain, it is also called the PLP-binding domain); a small N-terminal domain (Met1 to Gly44), which is predominantly formed by a long N-terminal loop and 3-stranded antiparallel β-sheet; and the C-terminal domain formed by five α-helices and a 2-stranded antiparallel β-sheet. Similar to other enzymes from this family, the active site of GenB2 is located in a deep cavity formed by a groove in the interface between the two protomers of the dimer, and each subunit contributes with essential residues for the substrate and cofactor binding (Figures 2 and S2). The contact interface between the two protomers has an area of 3300 Å^2^ and is predominantly formed by hydrophobic contacts (Figure 2).

Superposing the GenB2 structure with GenB1 and NeoB, we can observe that the overall structure is very much conserved, with rmsd values of 1.99 and 1.48 Å, respectively (Figure S3). GenB1 and NeoB also share sequence identities of about 29.6 and 36.1%, respectively, with GenB2. The main differences between these structures are in the extreme N-terminal loop, in the extreme C-terminal α-helix, and in a loop from residue 130 to residue 142 in GenB2 (Figure S3b). Interestingly, all these regions are involved in forming the substrate binding site cavity, and consequently, they should be involved in the specificity of the GenB2 for its substrates.

## Coenzyme Binding Site

**Figure 3:**
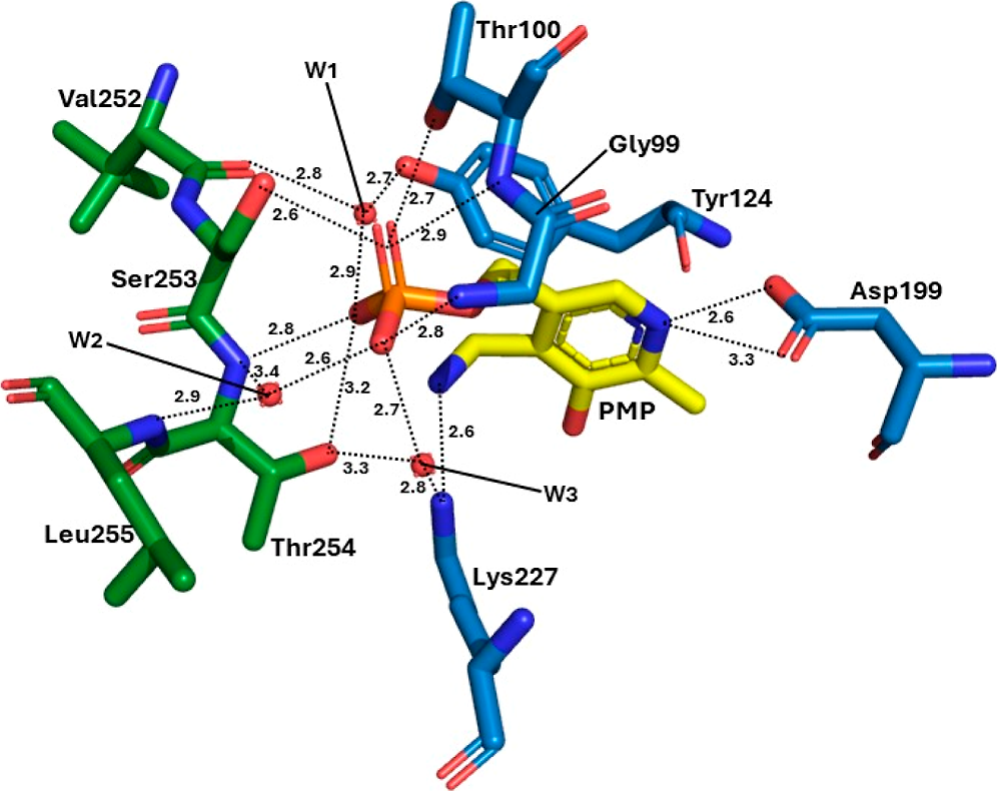
Coenzyme binding site of GenB2. The PMP is represented by carbons in yellow. Residues with carbons in blue are from protomer A, and residues with carbons in green are from protomer B. The red spheres named W1, W2, and W3 are water molecules. The traced lines are hydrogen bonds that involve the coenzyme, and the distances are represented in Å.

By the analysis of the electron density from the structures of GenB2 shown here, we cannot observe in any of them the formation of the Schiff base with the catalytic Lys227 (Figure S4). However, the coenzyme is eluted with GenB2 during purification since we can observe a strong pink color. Based on what has been reported previously,^23,33,34^ we have probably obtained the structure in complex with pyridoxamine 5-phosphate (PMP). Each dimer of GenB2 has two PMP molecules bound at the interfaces between the protomers. Residues of both protein molecules are responsible for anchoring the coenzyme in the binding site. The PMP molecule forms an extensive hydrogen bond network with protein residues. These residues include the critical catalytic residue Lys227, which interacts with the primary amine of the coenzyme, and the conserved (in type I PLP-dependent proteins) Asp199, which interacts with the nitrogen of the pyridine ring of PMP. Further interactions include an interaction of the main chain NH of Tyr124 with the pyridine nitrogen of PMP (all of these from the same protomer, here called A). Tyr124 also performs an edge-π interaction with the PMP ring. On the other hand, the phosphate moiety of PMP hydrogen bonds with residues of both protomers, including Gly99 and Thr100 from protomer A and Ser253 and Thr254 from protomer B. A water molecule also mediates hydrogen bonding between the phenolic OH of Tyr124 from protomer A and the main-chain oxygen of Val252 from protomer B. Another water molecule also mediates an interaction between the phosphate moiety with the main chains of Thr254 and Leu255 from protomer B. Finally, a third water molecule is involved in the interaction of the phosphate group of PMP with Lys227 from protomer A and Thr254 from protomer B (Figure 3).

When the binding mode of PMP is compared to those of the other PLP-dependent enzymes from gentamicin biosynthesis, particularly GenB1, GenB3, GenB4, or NeoB from neomycin biosynthesis, we can observe that, as expected, most of the interactions are conserved in all of these enzymes (Figure S5).

## Substrate-Binding Site

**Figure 4:**
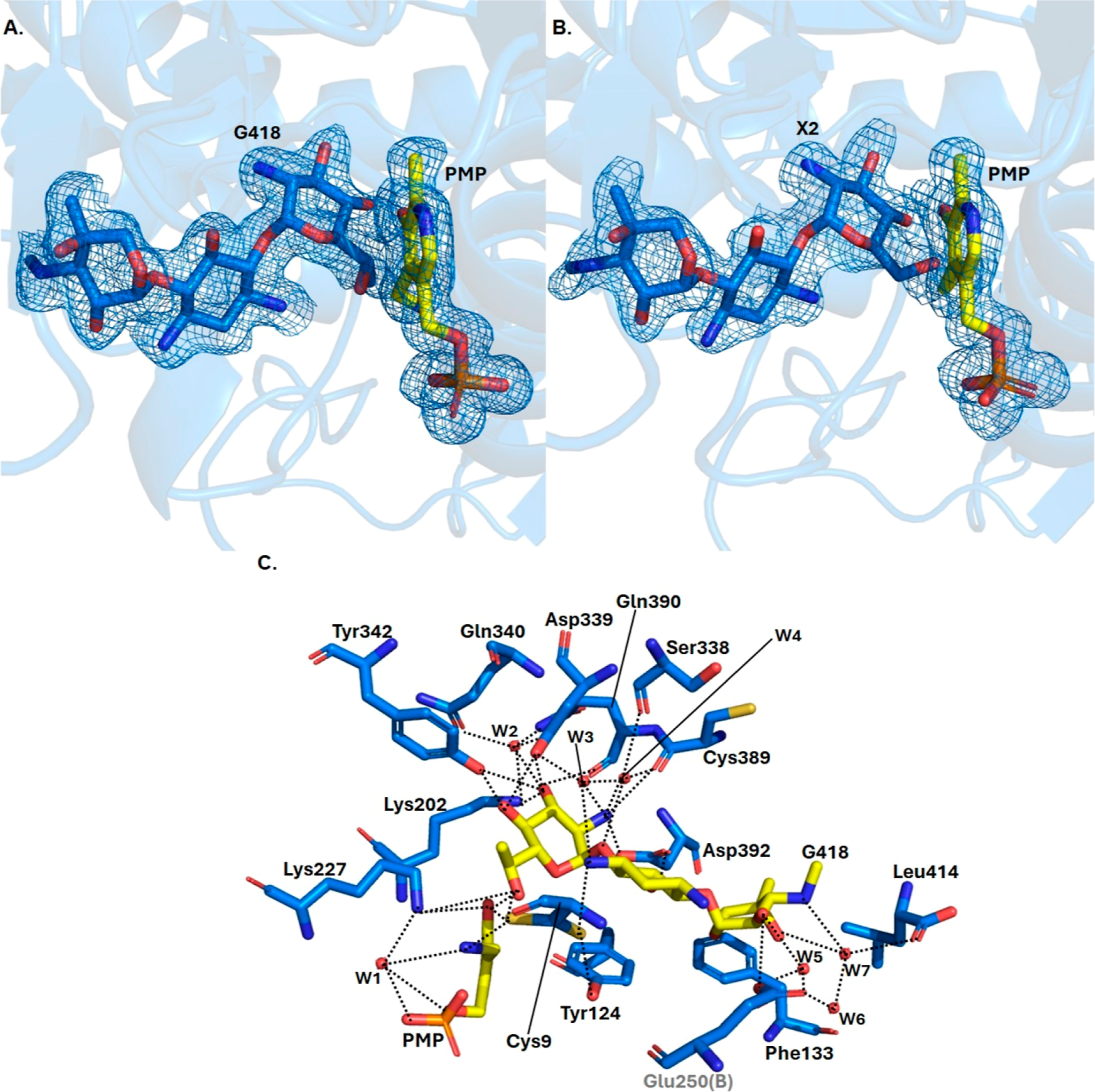
Complexes of GenB2 with substrate-like molecules. (A) Electron density contours for G418 and (B) gentamicin X2. The electron density contours were prepared based on a 2Fo–Fc map. G418 and gentamicin X2 are shown in blue with carbon atoms. Figures A and B also show the PMP, which has the carbon atoms in yellow. (C) Residues involved in the substrate binding in GenB2. The carbons in blue are those from GenB2, and the carbon atoms in yellow are from G418. Glu250(B) is an amino acid from chain B from the dimer of GenB2 and is labeled in gray. The dotted lines are hydrogen bonds between the GenB2 residues and G418. The red spheres are water molecules (W1–7). Two side chains for the Cys9 are shown because two conformations were observed in the electron density map during the refinement.

In order to gather insights into the binding mode of GenB2 substrate gentamicin C2a, we have obtained the structure of GenB2 in complex with gentamicin G418 and gentamicin X2 (Figure 4a,b). G418 is produced from gentamicin X2 in earlier steps of gentamicin biosynthesis through methylation at C-6′ by GenK. The hydroxyl group at C-6′ is then oxidized by GenQ to produce 6′-DOG, which is further transaminated by GenB1 to produce JI-20Ba. This molecule is then dideoxygenated by GenP and GenB3 to produce gentamicin C2a.^20,21^ It has been reported that GenB2 still retains some aminotransferase activity on 6′-DOG and is able to catalyze the epimerization reaction of the methyl group at C-6 of both JI20Ba (producing JI20Bb) and C2 to produce C2a.^24^ Based on that, it would be expected that G418 could be a good substrate analogueue for GenB2 since it is the same as JI20Ba but with a hydroxyl group instead of the amino group at position C-6′ (Figure 1).

G418 binds to a groove in GenB2, which is negatively charged and agrees with the complementary charge of a positively charged substrate (Figure S6). Also, several aromatic residues have face-to-face interactions with the sugar rings of the substrate. Thus, Tyr124 interacts with ring I, while Phe133 interacts with ring III. Additionally, a number of amino acid residues interact with the three different rings of the substrate analogues. Ring III, the most external ring of the substrate, is the only one to interact with the adjacent protomer (protomer B). This does not directly interact with ring III, but several interactions are mediated by water molecules (Figure 4c). The ring I interacts via hydrogen bonds with Cys9 and Asp392. Also, indirect contacts mediated by waters are observed with Ser338, Asp339, and Cys389 (Figure 4c). As expected, ring II, which is most deeply buried in the GenB2 structure, engages in a large number of direct and indirect interactions. It is possible to observe direct interactions with Lys227, Tyr342, Asp339, Lys202, Cys389, Gln390, and Asp392 and indirect interactions mediated by water molecules with Ser338, Gln340, Gln390, and Lys202 (Figure 4c). By analysis of the structure of GenB2 in complex with gentamicin X2, which lacks the C-6′ methyl group, we observe that most of the interactions are conserved and that the presence or absence of the methyl group does not significantly affect the binding (Figure S7).

Additionally, when we superpose the structure of holo GenB2 with holo GenB2 in complex with G418, we can observe that the substrate does not cause any significant conformation changes in the region of the substrate binding site (Figure S8). This also has been reported for other PLP-dependent enzymes involved in gentamicin biosynthesis.^21,23,25^ The most significant change between these two structures occurs in the extreme N-terminal loop, particularly from Ala7 to Thr10 (Figure S8). Interestingly, Cys9 in the complex of holo GenB2 with G418 adopts a double conformation in our structure in order to interact with the substrate. Also, other minor changes are observed in the side chains of amino acids, which optimize the interaction with the substrate and might play an important role in the catalytic mechanism of the enzyme. Although GenB2 is highly similar to GenB1 or NeoB, the extreme N-terminal loop seems not to undergo significant conformational changes in the binding of the substrate. In addition, in GenB1 and NeoB, this region, despite having a conserved cysteine residue (Figure S1), is not close enough to perform any contact with the aminoglycoside-like substrates (Figure S9a). However, by superposing GenB2 with GenB1, we observed that the binding modes of the substrates in these two enzymes are very similar, consistent with the residual aminotransferase activity of GenB2 (Figure S9b).

### Active Site and Mechanism of Catalysis

**Figure 5:**
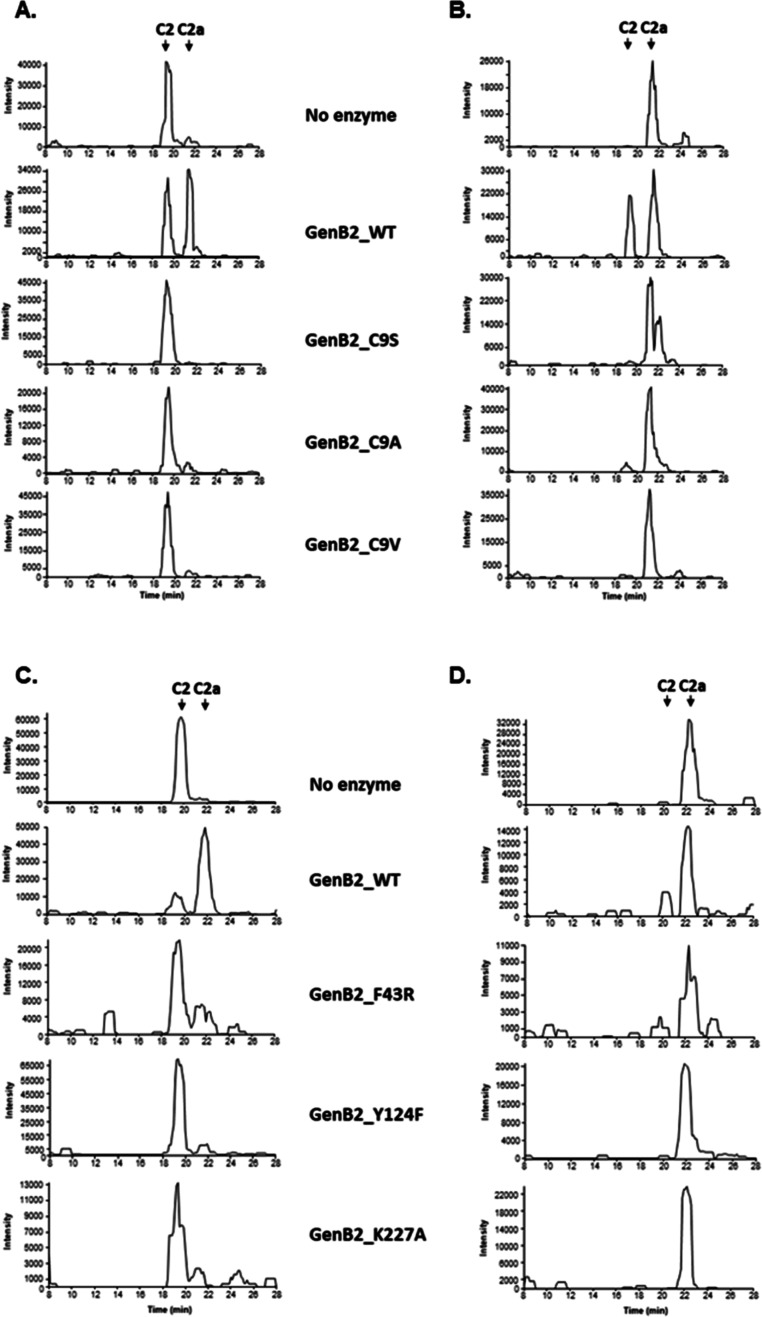
Activity assays of wild-type GenB2. Mutants for Cys9 mutants (C9S, C9A, and C9 V) (A) gentamicin C2 as the substrate; (B) gentamicin C2a as the substrate. Activity assays of GenB2 and its F43R, Y124F, and K227A mutants. (C) Gentamicin C2 as the substrate; (D) gentamicin C2a as the substrate. All original traces of this experiment are shown in Figure S10.

PLP-dependent enzymes can catalyze a number of different reactions based on the plasticity of the cofactor PLP. In the case of those reactions that involve the amination of sugars, keto sugars usually serve as substrates. The structure of GenB2 reveals, in the active site, the presence of a conserved aspartic residue that contributes to maintaining and stabilizing the protonated, positively charged pyridinium ring of the cofactor. Based on that, it is no surprise that GenB2 is able to catalyze an aminotransferase reaction converting 6′-DOX and 6′-DOG in JI-20A and JI-20Ba, respectively,^20^ in the presence of donor amino acids since all residues involved in transamination reactions are present. However, GenB2 is the enzyme that catalyzes the epimerization of gentamicin C2a into C2, and consequently, further amino acid residues of the protein should play this role. Based on that, we have carried out site-directed mutagenesis of potential residues of the active site that could be involved in the epimerization reaction. We chose those amino acids that should play a role in stabilizing the position of the substrate in the active site or that could play a direct role in catalysis. Site-directed mutagenesis was performed on Cys9 since its position allows contact with both the coenzyme and C-6′-methyl group of the substrate. We constructed the mutants C9S, C9A, and C9 V. Additionally, we also constructed the mutants F43R, Y124F, and K227A. All of the protein mutants were recombinantly produced and purified, and their epimerization activity was assayed against purified gentamicin C2 and C2a according to the experimental procedures. Figure 5 shows that the wild-type GenB2 was able to perform a high ratio of conversion of gentamicin C2 into C2a or C2a into C2 (Figure 5b), in comparison with the absence of enzyme. On the other hand, all the constructs with amino acid substitution of C9 rendered little or no conversion (Figure 5a), strongly indicating that this amino acid is crucial for the catalytic activity of GenB2. In addition, K227A, as expected, completely abolished the epimerization reaction. For the other two studied mutations involving aromatic amino acids, F43 and Y124, we also can observe that Y124F strongly impacts the epimerization reaction (Figure 5c,d). Consequently, the phenol group of Y124 also plays an important role in the catalysis of GenB3. In contrast, the substitution of F43 for an arginine decreased the conversion of the products in comparison to the wild-type enzyme, but its impact was minor in comparison to those of the other studied mutations.

**Figure 6:**
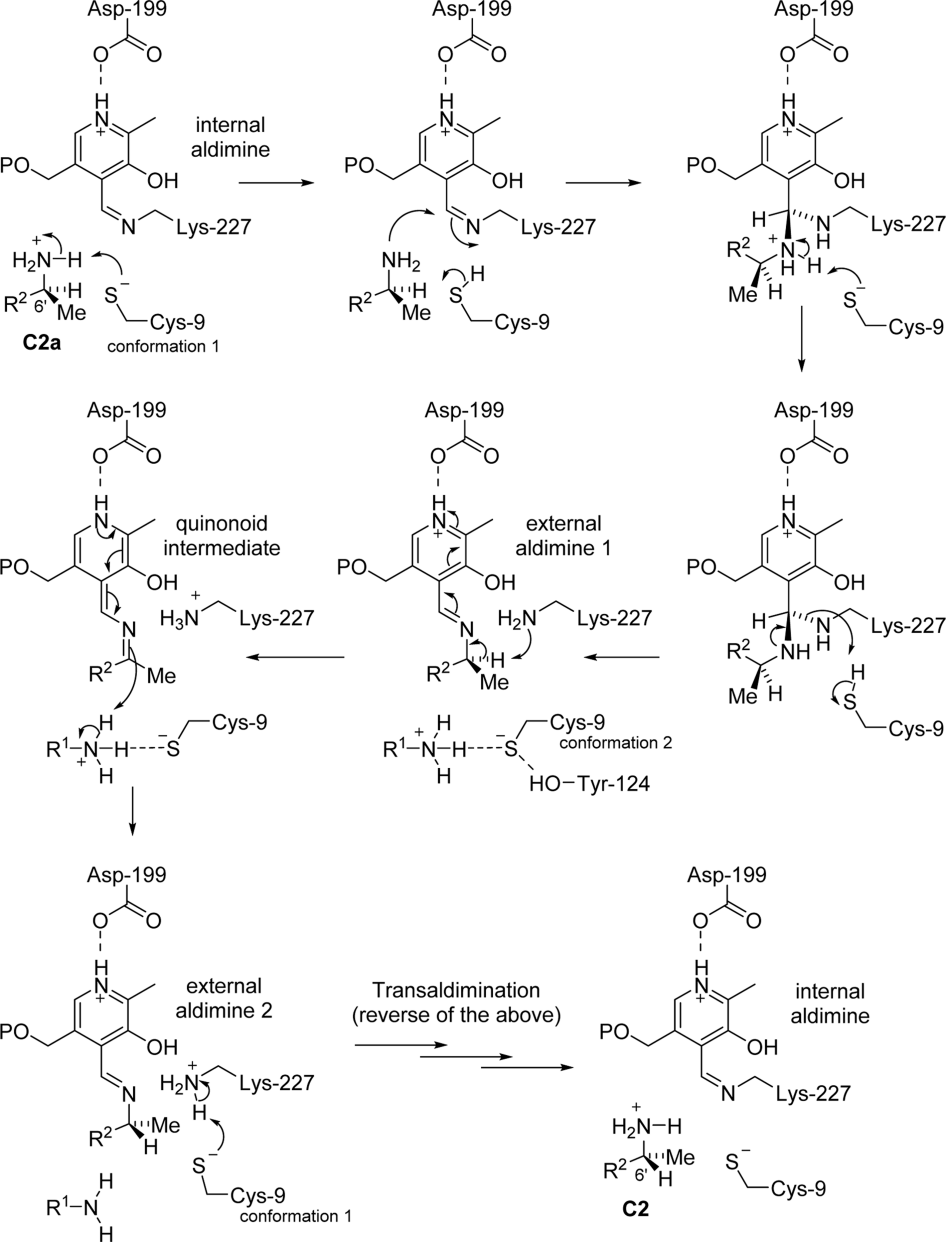
Proposed catalytic mechanism for GenB2 starting from the resting state of GenB2 (internal aldimine) and gentamicin C2a. Cys9 acts as the proton shuttle that ensures the amino groups involved, the C-6′ amino group, the ε-amino group of Lys227, and the C-4 amino group (which acts as an acid/base group), can be in the right protonation state. R1 = ring I (C-4); R2 = ring II (C-5′).

Based on our structural and functional study of GenB2, we can propose a catalytic mechanism for the epimerase reaction catalyzed by this PLP-dependent enzyme (Figure 6), in which the Cys9 has a crucial role in the common mechanisms of deprotonation/reprotonation involved in epimerases.^35^ It is proposed that the acid/base groups on either side, responsible for deprotonating the external aldimines and then reprotonating the quinonoid intermediate, are the ε-amino group of Lys227 (which is expected in PLP-dependent enzymes) and the amino group attached to C-3 of the 2-DOS ring (ring I), which is the only group appropriately placed to be the acid–base group on the opposite side from Lys227. Additionally, Asp199, which is a conserved residue in PLP-dependent enzymes from fold I, is involved in maintaining the protonated state of the pyridine ring of PLP through its interaction with N1. Cys9 acts as a proton shuttle that ensures that each amino group is in the correct protonation state for the next step of the mechanism.

Despite having some similarities, the mechanism proposed for GenB2 is unprecedented and distinct from the other PLP-dependent racemases, including the alanine racemase family.^36^ PLP-dependent racemases belong to family III of the PLP-dependent fold, distinct from family I, to which GenB2 belongs. In the case of PLP-dependent racemases, a tyrosine, together with the essential lysine residue, plays critical roles in the two-base mechanism for protonation/deprotonation of the substrate, acting as enantiospecific Brønsted bases to remove the α-proton from the l-alanine substrate.^35,37,38^

Our ligand-bound crystal structures show that the thiol of Cys9 in conformation 1 can hydrogen bond to the C-6′ amino group of gentamicin C2a and could, in its deprotonated form, be the base that deprotonates this initially protonated amine, allowing it to nucleophilically attack the internal aldimine (Figure 6). Subsequently, the thiol of Cys9 could potentially be the acid that protonates the e-amine of Lys227, allowing it to leave to form the first external aldimine. Once the external aldimine is formed, Cys9 can change to conformation 2, where it hydrogen bonds with the C-4 amino group on ring I and Tyr124. The ε-amine of Lys227 can then act as the base, giving the quinonoid intermediate, and the protonated amino group attached to C-4 could then protonate from the opposite face, giving the epimeric gentamicin C2 external aldimine. Cys9 could then revert to conformation 1 and act as the proton shuttle in the reverse transaldimination that converts the second external aldimine back to the internal aldimine and releases gentamicin C2. In the reverse direction, the deprotonated thiol of Cys9 might act as the base to deprotonate the protonated amino group attached to C-4 to act as a base.

## Summary

In summary, we have determined the structure of the PLP-dependent enzyme GenB2 in holo form and in complex with two substrate analogues, G418 and gentamicin X2. Based on the structural analysis, we could confirm that GenB2 has distinct epimerase activity inside the type I PLP-dependent enzyme fold family that has so far not been described for this class of enzymes. A cysteine residue near the N-terminus, despite being present in other paralogous enzymes, including GenB1 and NeoB, adopts a catalytic role in GenB2 and plays an essential role in the epimerase activity of GenB2. This investigation expands the range of versatilities of PLP-dependent enzymes.

## Methods

### Cloning, Expression, and Purification

The cloning, expression, and purification were performed according to Guo et al., 2014,^20^ with minor modifications. Briefly, the encoding region of GenB2 was inserted in a pET28a plasmid, and expression was carried out in BL21(DE3) using 0.1 mM isopropylthiogalactoside (IPTG) at 16 °C overnight. For the purification, the cells were resuspended in a buffer containing 50 mM Tris-HCl, pH 7.8, and 200 mM NaCl (buffer A) and disrupted by sonication. The soluble and insoluble fractions were separated by centrifugation. The soluble fraction was passed through an IMAC 5 mL column charged with nickel. The protein was eluted using a linear gradient of buffer A containing 500 mM imidazole in an Akta Purifier (Cytiva). For sample polishing, GenB2 was further purified by molecular exclusion chromatography using a gel filtration column 16/60 200 (Cytiva). The sample containing the protein of interest was concentrated and stored at −80 °C.

### Site-Directed Mutagenesis

The NotI-*Bam*HI fragment from the genB2/pET28 plasmid construct,^20^ which contains the entire wild-type genB2 gene plus an extra 57 nucleotides at the 5′-end, was recloned into an MN31–1 vector (Eurofin) between the NotI and *Bam*HI sites. The resulting plasmid genB2/MN31-1 was used as a template for QuickChange site-directed mutagenesis using the following primer pairs: pB2_C9S_F 5′-caacgctgacggttccacgccgtac-3′/pB2_C9S_R 5′-gtacggcgtggaaccgtcagcgttg-3′; pB2_C9 V_F 5′-caacgctgacggtgtcacgccgtac-3′/pB2_C9 V_R 5′-gtacggcgtgacaccgtcagcgttg-3′; pB2_C9A_F 5′-caacgctgacggtgccacgccgtac-3′/pB2_C9A_R 5′-gtacggcgtggcaccgtcagcgttg-3′. The QuickChange PCR was carried out with 30 cycles of denaturation at 98 °C for 10 s, annealing at 60 °C for 30 s, and extension at 72 °C for 4 min plus a final extension at 72 °C for 10 min using Pfu DNA polymerase. 0.5 μL of Dpn I was then added to digest the template at 37 °C for 1 h. 1 μL of the mixture was then used to transform *E. coli* NovaBlue cells. The plasmids isolated from the transformants were sequenced using primer pairs pEX-For 5′-ggagcagacaagcccgtcagg-3′/pEX-Rev 5′-aggctttacactttatgcttccggc-3′ to confirm the mutations. The plasmids with desired mutations were digested with N*de*I and *Bam*HI, purified by gel extraction, and inserted into plasmid pET28a(+). The resulting constructs were verified by DNA sequencing with a T7–T7t primer pair.

### Protein Crystallization, Structure Determination, and Analysis

Holo GenB2 at 8 mg mL^–1^ in buffer A was crystallized using a condition composed of 0.1 M PIPES, pH 6.0, 1 M NaCl, 29% PEG 4000, and 30% 6-aminohexanoic acid using Limbro plates in drops containing a 1:1 ratio of protein solution and crystallization condition. GenB2 in different complexes was crystallized under the same condition but with GenB2 previously incubated with at least 100 mM of gentamicin X2 or G418. Small plate-like crystals appeared after 2 days at 18 °C. Crystals of holo GenB2 or in complex with ligands were suspended in a cryogenic solution comprising 30% glycerol and 70% crystallization well solution. They were harvested by using nylon loops and quickly frozen in liquid nitrogen.

The X-ray data collection of GenB2 crystals was performed at Sirius, Manacá beamline, CNPEM, Brazil. The data was processed using XDS^39^ and scaled using AIMLESS^40^ from the CCP4 suite.^41^ The structure of holo GenB2 was solved by molecular replacement using the program Phaser^42^ from Phenix suite^43^ and the structure of GenB1 (PDB entry 5Z83) as a probe. Further structures in complex with the different ligands were also determined by molecular replacement using the holo GenB2 structure. The crystallographic refinement was carried out using Phenix.refine,^44^ and the real space and visual inspection refinement was carried out using Coot.^45^ The stereochemistry quality of the structure was checked using Molprobity.^46^ The figures were prepared using PyMOL (The PyMOL Molecular Graphics System, Version 1.2r3pre, Schrödinger, LLC).

### Enzymatic Assay

The isomerase activity of the wild type and mutants of GenB2 was performed according to Guo et al., 2014.^20^ Briefly, the wild type and mutant enzymes at 20 μM in 50 mM Tris-HCl, pH 8.0 buffer were incubated in the presence of substrates gentamicin C2 or C2a at 200 μM. The reaction was maintained overnight at 30 °C and quenched by the addition of 100 μL of chloroform. The mixture was vortexed and centrifuged for protein removal, and the supernatant was analyzed by HPLC.

## Supporting Information Available

Data collection and refinement statistics; sequence alignment of different PLP-dependent enzymes involved in the biosynthesis of gentamicin and neomycin; residues involved in the dimerization of GenB2; superposition of GenB2 with orthologous proteins; electron density contours for the catalytic Lys227 and the coenzyme; coenzyme (PMP) binding site conservation; substrate binding site of GenB2; conservation of the binding mode of G418 and gentamicin X2 in the binding site of GenB2; superposition of GenB2 at different complexes; binding mode of substrates and/or analogues on PLP-dependent enzymes from the biosynthesis of aminoglycosides; and LC–MS original traces for the epimerase activity of GenB2 wild type and mutants (PDF)

## Abbreviations

BGC: biosynthetic gene cluster
PLP: pyridoxal 5′-phosphate
2-DOS: 2-deoxystreptamine
IPTG: isopropylthiogalactoside
PIPES: 1,4-piperazinediethanesulfonic acid
PEG: polyethylene glycol
PMP: pyridoxamine 5-phosphate
