# Supplementary material for: Structural and Functional Basis of GenB2 Isomerase Activity from Gentamicin Biosynthesis
Source: ACS Chem Biol. 2024 Aug 29;19(9):2002–11. doi: 10.1021/acschembio.4c00334 (PMC11420954; doi:10.1021/acschembio.4c00334)

# Supplementary Material

# The structural and functional basis of GenB2 isomerase activity from gentamicin biosynthesis

Gabriel Stephani de Oliveira[1#], Priscila dos Santos Bury[1#], Fanglu Huang[2], Yuan Li[3], Natália Cerrone de Araújo[1], Jiahai Zhou[4], Yuhui Sun[3], Finian J. Leeper[5], Peter F. Leadlay[2], Marcio Vinicius Bertacine Dias[1*]

1 Department of Microbiology, Institute of Biomedical Sciences, University of Sao Paulo, 05508000, Brazil.

2 Department of Biochemistry, University of Cambridge, Cambridge, CB21GA, UK.

3 Key Laboratory of Combinatorial Biosynthesis and Drug Discovery (Ministry of Education), and School of Pharmaceutical Sciences, Wuhan University, Wuhan 430071, China

4 State Key Laboratory of Quantitative Synthetic Biology, Shenzhen Institute of Synthetic Biology, Shenzhen Institute of Advanced Technology, CAS, Shenzhen 518055, China

5 Yusuf Hamied Department of Chemistry, University of Cambridge, Cambridge, CB2 1EW, UK

* Corresponding author: mvbdias@usp.br

# These authors contributed equally

**Supplementary Table 1**. Data collection and refinement statistics.

| | GenB2-holo | GenB2:G418 | GenB2:X2 |
|---|---|---|---|
| PDB entries | 9AUE | 9AU3 | 9B0C |
| Synchrotron source | Sirus-Brazil | Sirius-Brazil | Petra-III-Germany |
| Wavelength (Å) | 0.977180 | 0.977180 | 0.976250 |
| Resolution range (Å) | 39.59 -1.46 (1.51-1.46) | 39.52 -1.35 (1.4 -1.35) | 46.64-1.66 (1.72 -1.66) |
| Space group | C 2 2 21 | C 2 2 21 | C 2 2 21 |
| Unit cell (Å) | 75.36 103.75 104.12 | 75.14 103.05 104.13 | 75.41 104.19 104.65 |
| Total reflections | 944934 (94858) | 1170127 (111629) | 462308 (44735) |
| Unique reflections | 70268 (6986) | 88623 (8757) | 48785 (4780) |
| Multiplicity | 13.4 (13.9) | 13.2 (12.7) | 9.5 (9.4) |
| Completeness (%) | 99.98 (99.93) | 99.97 (99.87) | 99.64 (99.15) |
| Mean I/sigma(I) | 11.91 (1.16) | 14.83 (1.05) | 14.73 (1.91) |
| Wilson B-factor ($Å^2$) | 17.21 | 17.16 | 20.74 |
| R-merge | 0.13 (2.62) | 0.09 (2.12) | 0.11 (1.62) |
| R-meas | 0.13 (2.72) | 0.09 (2.21) | 0.12 (1.71) |
| R-pim | 0.04 (0.72) | 0.03 (0.62) | 0.04 (0.55) |
| CC1/2 | 0.999 (0.545) | 0.999 (0.568) | 0.999 (0.608) |
| CC* | 1 (0.84) | 1 (0.851) | 1 (0.87) |
| Reflections used in refinement | 70887 (6981) | 88605 (8746) | 48778 (4780) |
| Reflections used for R-free | 1995 (196) | 1997 (198) | 2439 (251) |
| R-work | 0.151 (0.301) | 0.14 (0.3) | 0.165 (0.273) |
| R-free | 0.197 (0.349 | 0.166 (0.338) | 0.191 (0.336) |
| CC(work) | 0.940 (0.667) | 0.978 (0.829) | 0.959 (0.686) |
| CC(free) | 0.910 (0.596) | 0.980 (0.840) | 0.950 (0.594) |
| Number of non-hydrogen atoms | 3693 | 3677 | 3611 |
| macromolecules | 3169 | 3161 | 3192 |
| ligands | 25 | 52 | 50 |
| solvent | 499 | 464 | 369 |
| Protein residues | 415 | 414 | 414 |
| RMS(bonds) | 0.006 | 0.020 | 0.007 |
| RMS(angles) | 0.87 | 0.87 | 0.90 |
| Ramachandran favored (%) | 99.03 | 98.54 | 98.06 |
| Ramachandran allowed (%) | 0.97 | 1.46 | 1.70 |
| Ramachandran outliers (%) | 0.00 | 0.00 | 0.24 |
| Rotamer outliers (%) | 0.30 | 1.20 | 1.49 |
| Clashscore | 2.54 | 3.95 | 4.19 |
| Average B-factor ($Å^2$) | 25.58 | 28.52 | 25.17 |
| macromolecules | 23.52 | 25.10 | 23.74 |
| ligands | 31.13 | 60.36 | 36.84 |
| solvent | 38.38 | 48.28 | 36.02 |

Statistics for the highest-resolution shell are shown in parentheses.

**Supplementary figure 1. Sequence alignment of different PLP-dependent enzymes involved in the biosynthesis of gentamicin and neomycin. * represents the conserved residues involved in the cofactor binding site.**

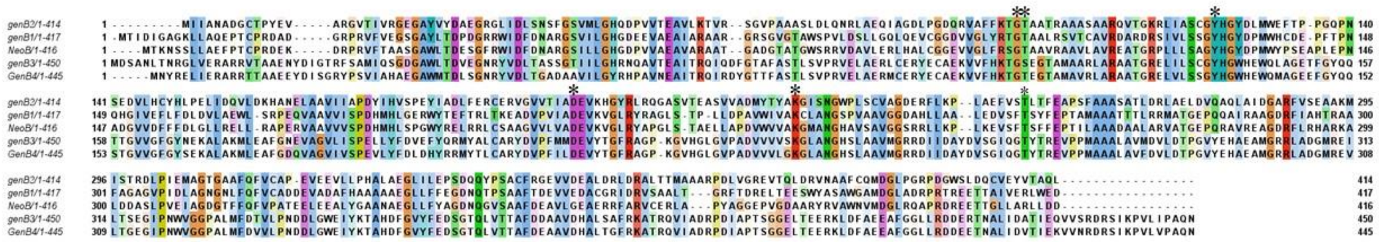

**Supplementary Figure 2. Residues involved in the dimerization of GenB2.**

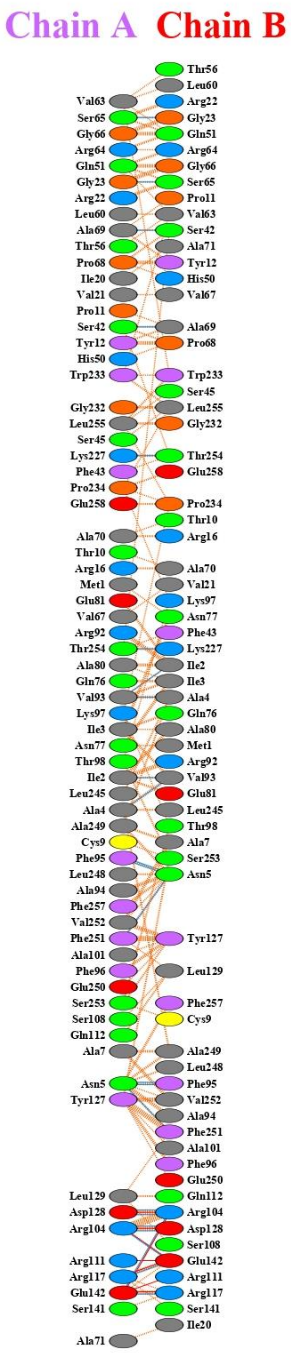

**Supplementary figure 3. Superposition of GenB2 with orthologous proteins.** GenB2 is represented in yellow, GenB1 is represented in blue and NeoB in green.

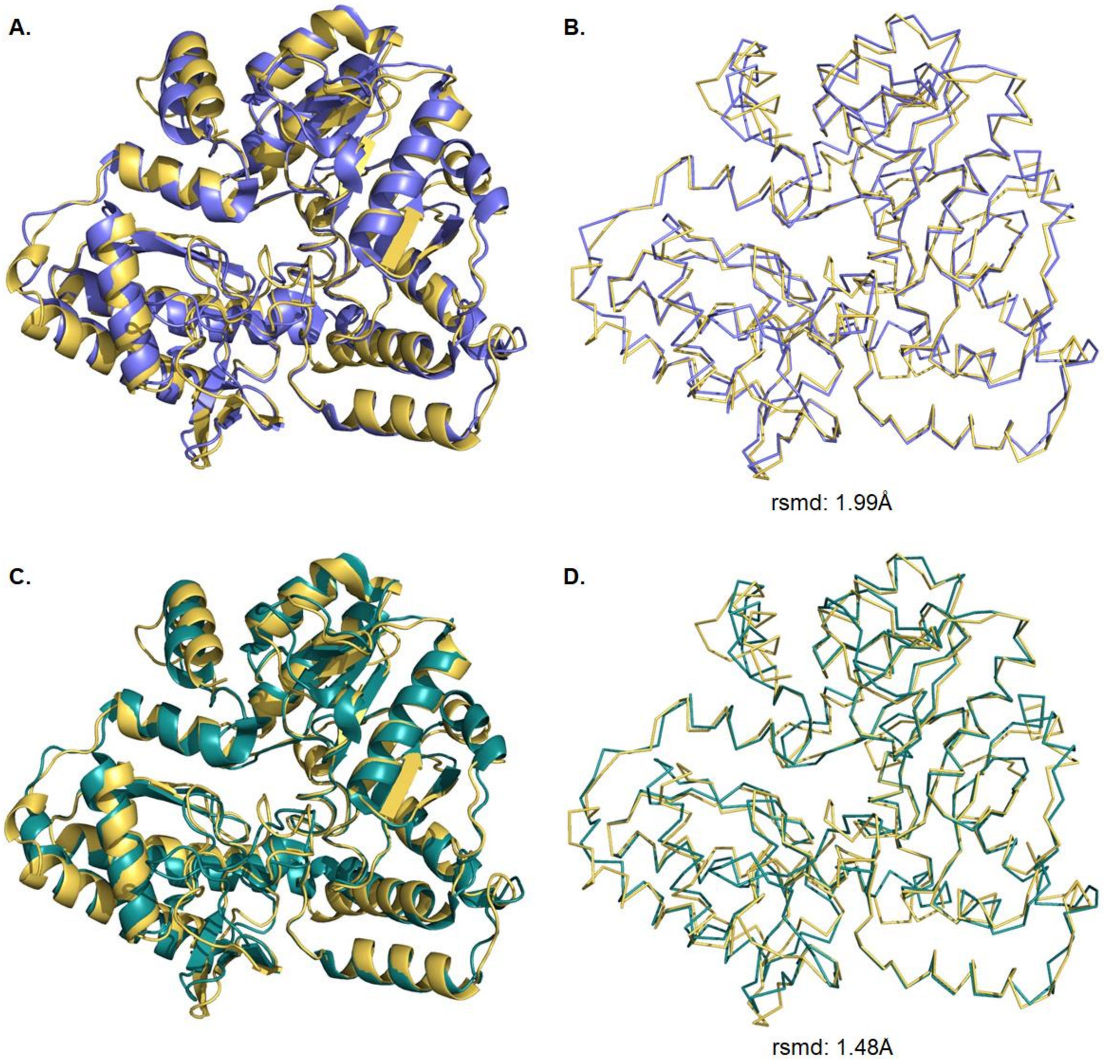

**Supplementary figure 4. Electron density contours for the catalytic Lys227 and the coenzyme.** The figure shows that there is no formation of Schiff base between the Lys227 and the coenzyme and then we assumed that the coenzyme bound in the structure is PMP.

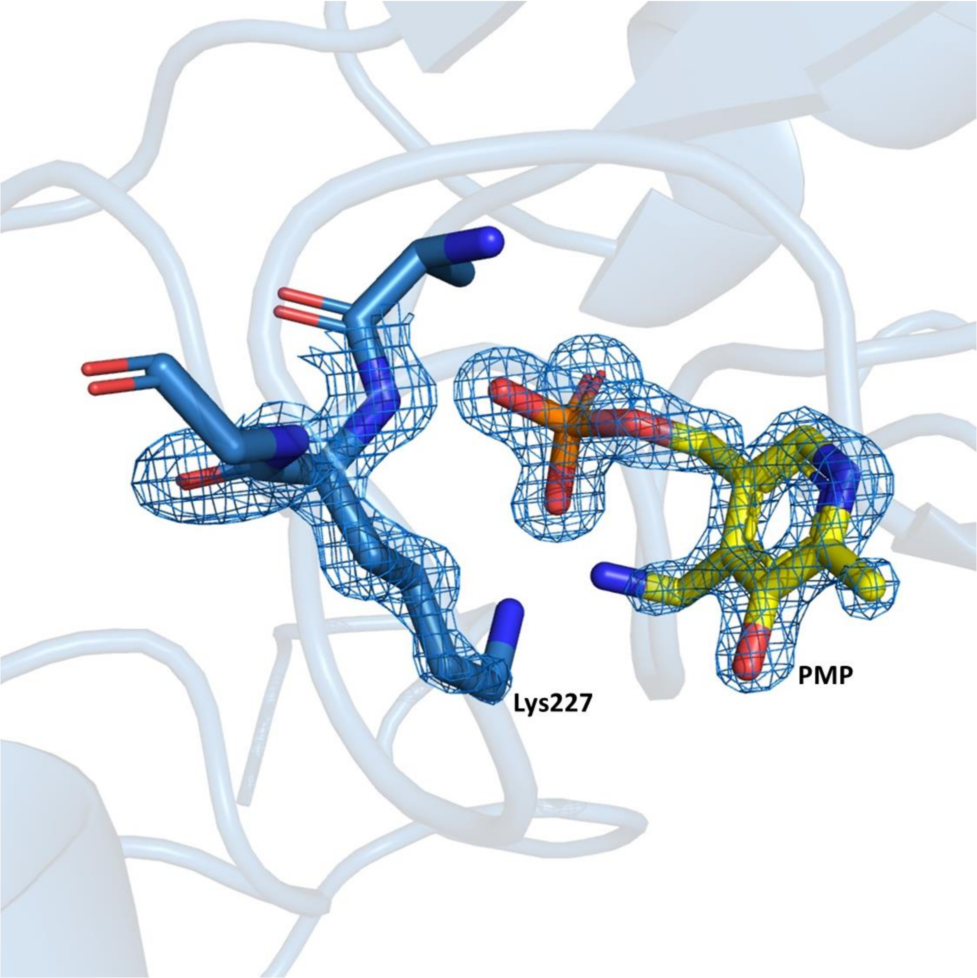

**Supplementary figure 5. Coenzyme (PMP) binding site conservation.** The superposition of GenB2 (Blue), GenB1 (pink), GenB3 (yellow), GenB4 (green), and NeoB (orange) shows that the residues involved in the PMP binding site are conserved, with a few exceptions, into the different PLP-dependent enzymes from aminoglycoside (gentamicin and neomycin) biosynthesis.

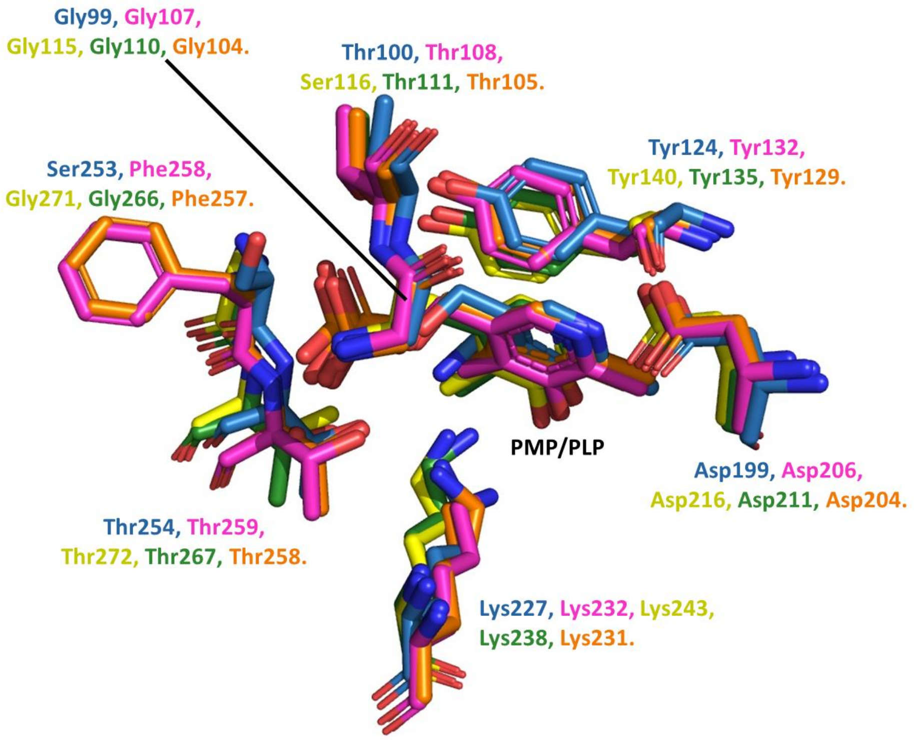

**Supplementary Figure 6. Substrate binding site of GenB2.** The substrate binding site shows a predominance of negative charge, which is complementary to the anionic charges of the substrate. In the figure is shown the binding mode of G418, which is represented in stick and with carbon atoms in blue**.**

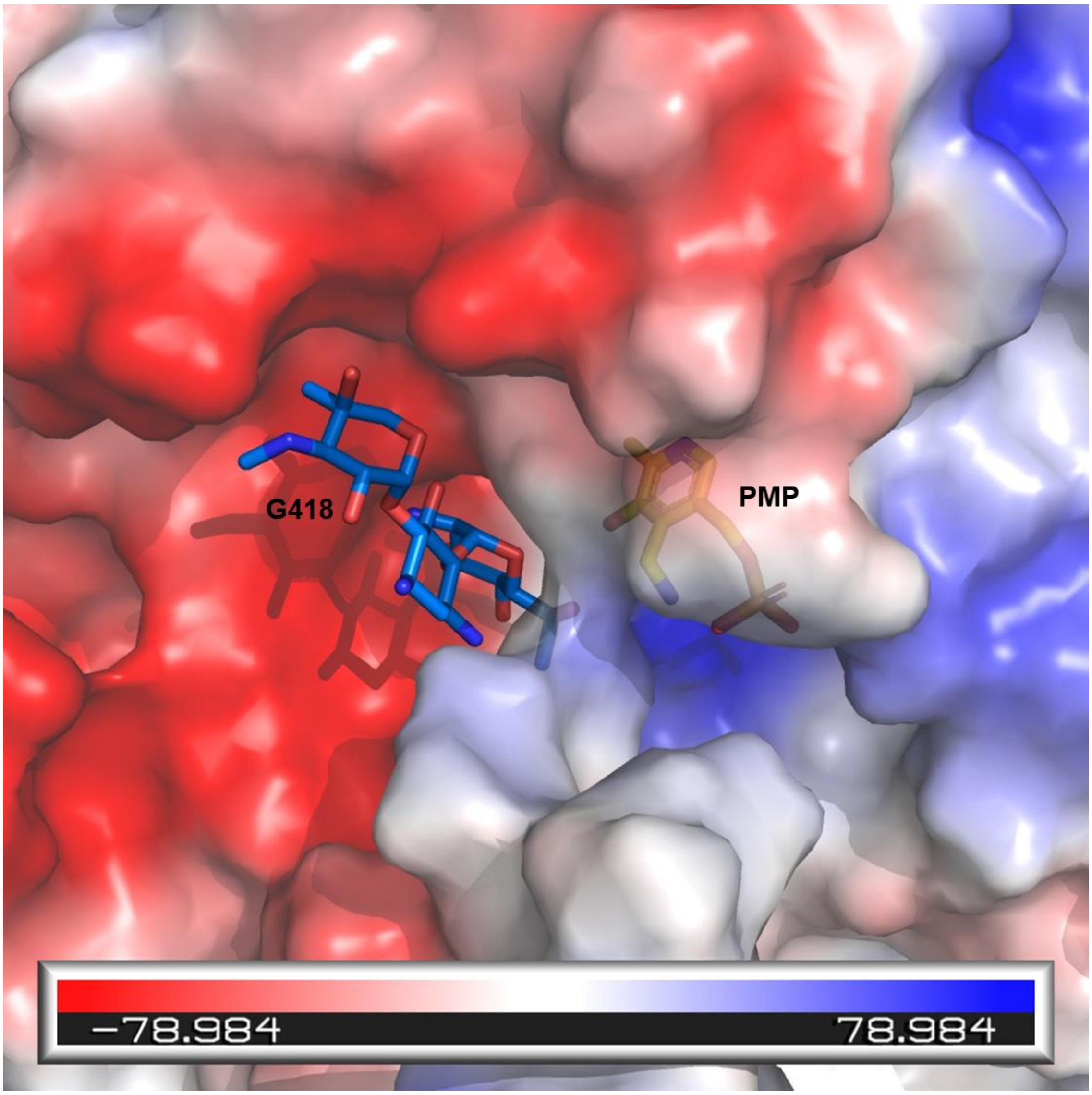

**Supplementary figure 7. Conservation of the binding mode of G418 (orange) and Gentamicin X2 (green) in the binding site of GenB2.** The carbons in blue sticks are from the GenB2 in complex with G418, while the carbons in yellow sticks are from the complex with Gentamicin X2. The major difference is observed in the region of the Cys9 that is present in the extreme N-terminal loop, and it is a flexible region, although Cys9 is proposed to be part of the catalytic mechanism of GenB2.

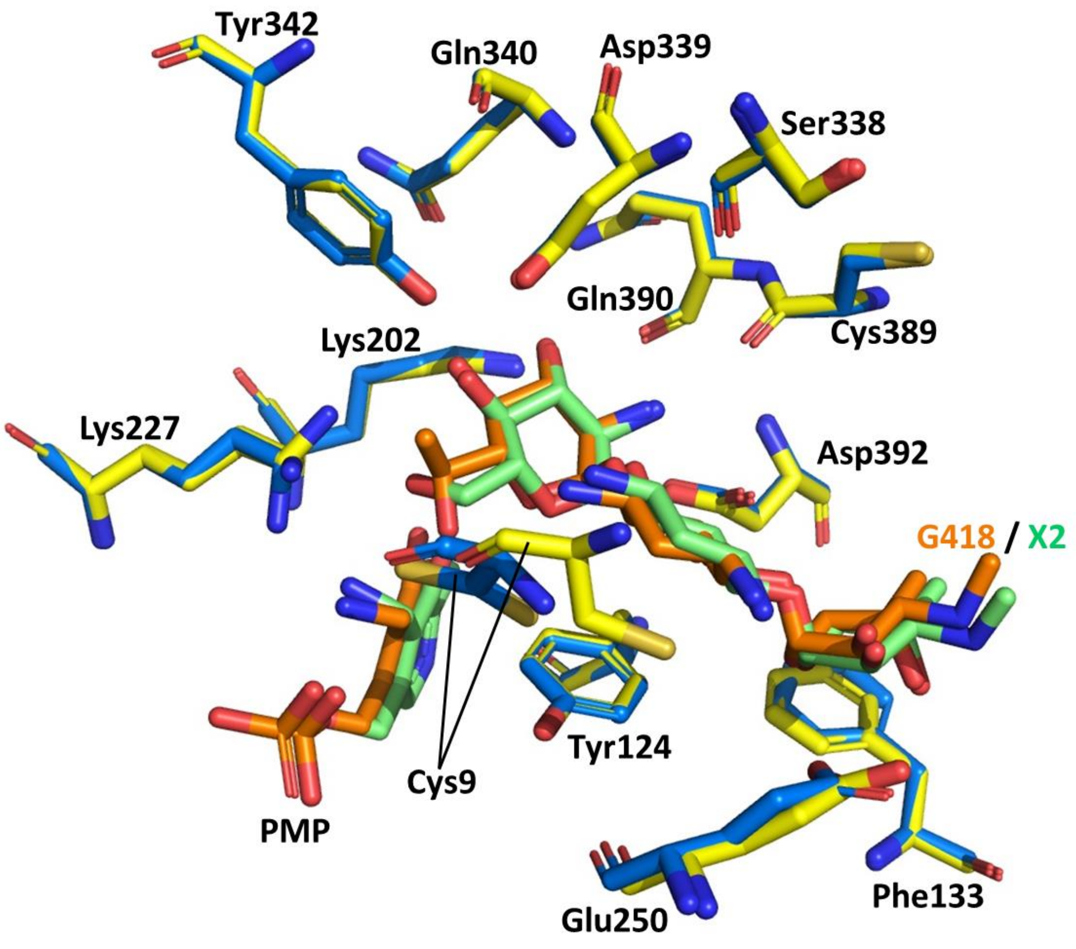

**Supplementary Figure 8. Superposition of GenB2 at different complexes.** In green is shown GenB2 in its holo form (it means in complex with PMP) and in Blue is shown GenB2 in complex with PMP and G418. The major difference between the two structures is observed in the extreme N-terminal region, particularly in the position of Cys9, which is involved in the catalytic mechanism of GenB2 (here represented by an arrow).

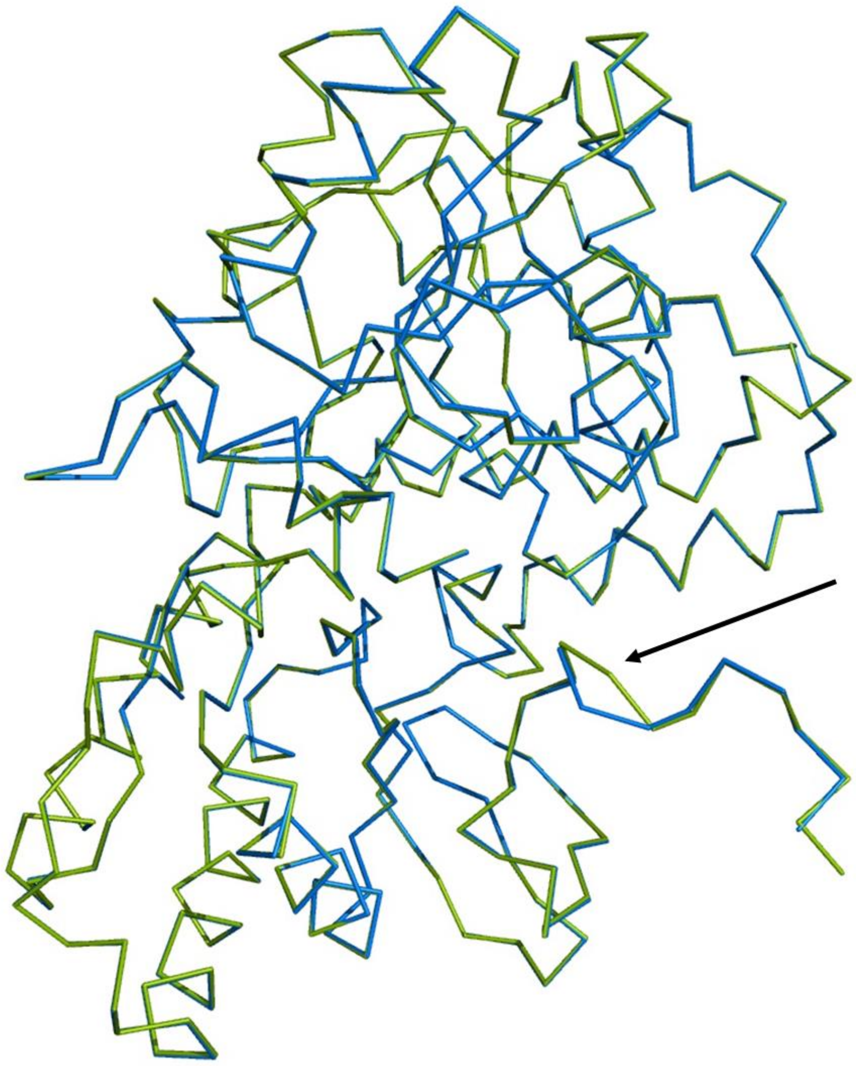

**Supplementary figure 9. Binding mode of substrates and/or analogs on PLP-dependent enzymes from the biosynthesis of aminoglycosides.** (A) Superposition of GenB2 in complex with PMP and G418 (yellow) and GenB1 in complex with external aldimine (blue) and (B) Superposition of GenB2 in complex with PMP and G418 (yellow) and NeoB in complex with external aldimine (green).

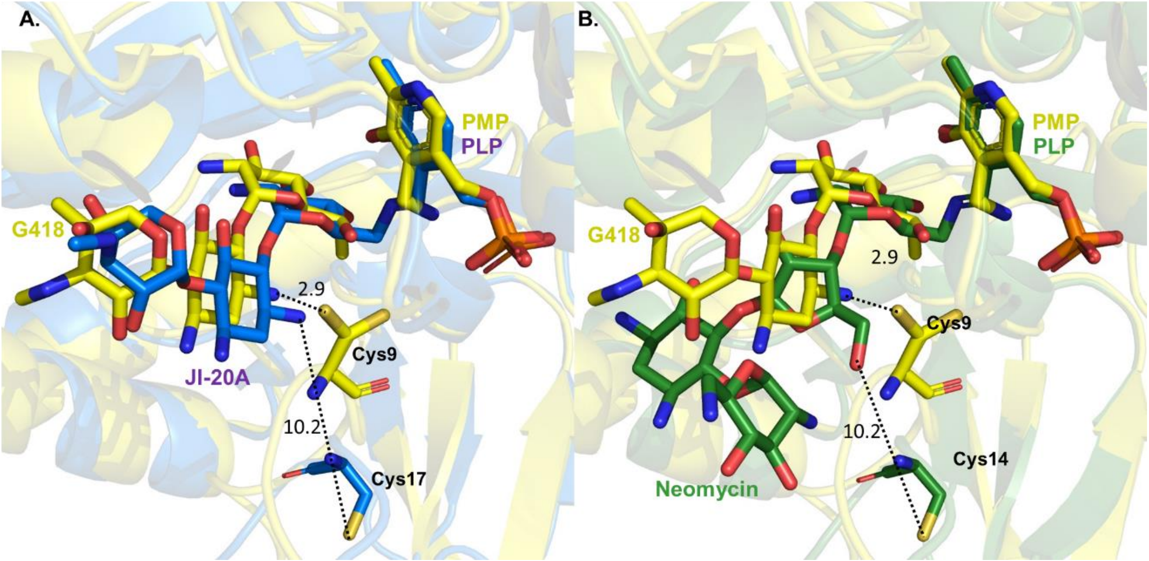

**Supplementary figure 10.** Original LC-MS EIC extraction traces of assays assessing the epimerase activity of WT GenB2 and its mutants (.

### A. Assays of Gentamicin C2 with GenB2_WT, _C9S, _C9A, _C9V

A1. Gentamicin C2 without enzyme

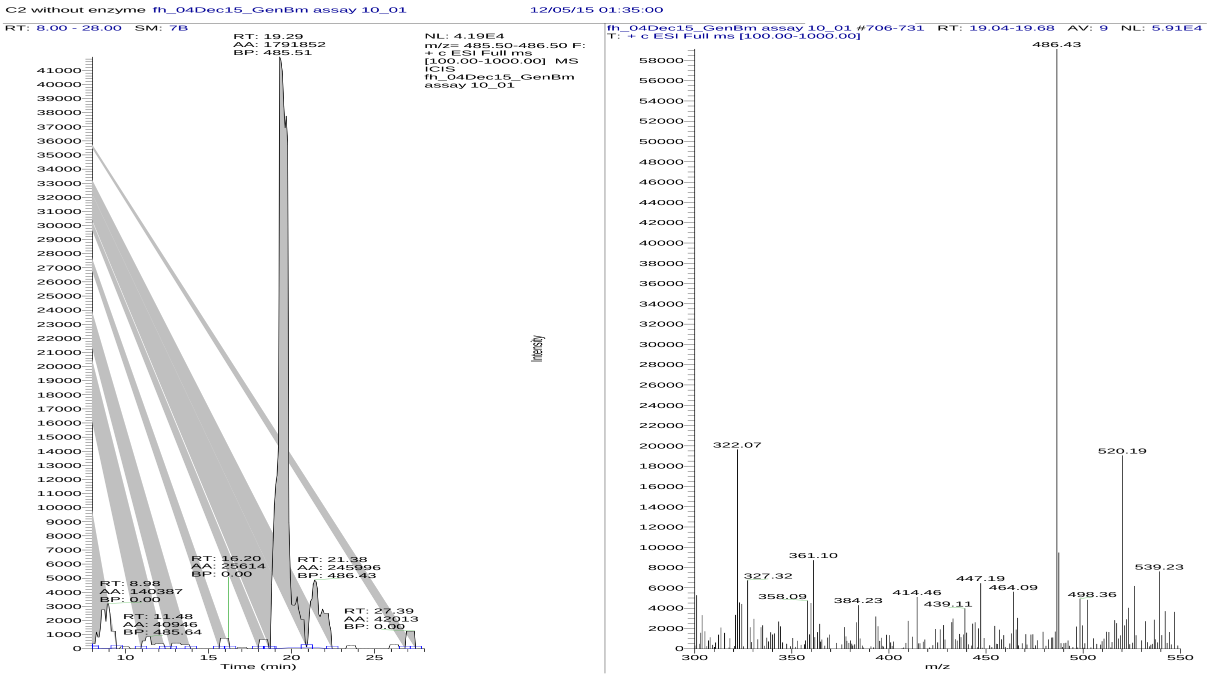


A2. Gentamicin C2 + GenB2-WT

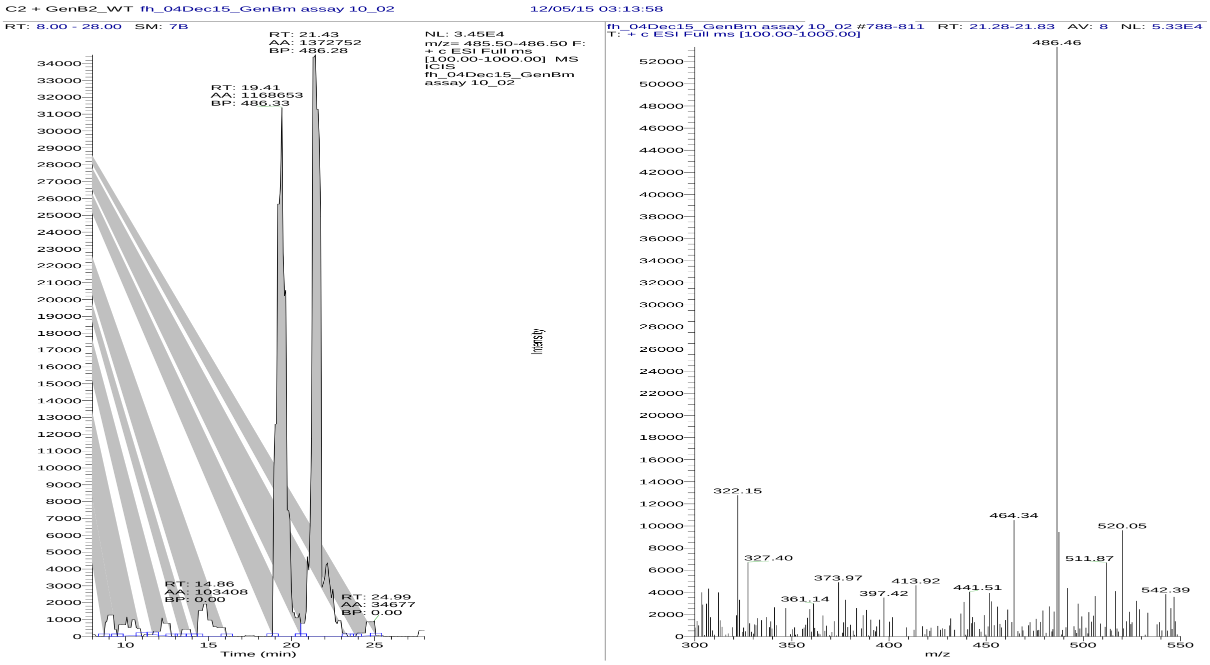

## A3. Gentamicin C2 + GenB2_C9S

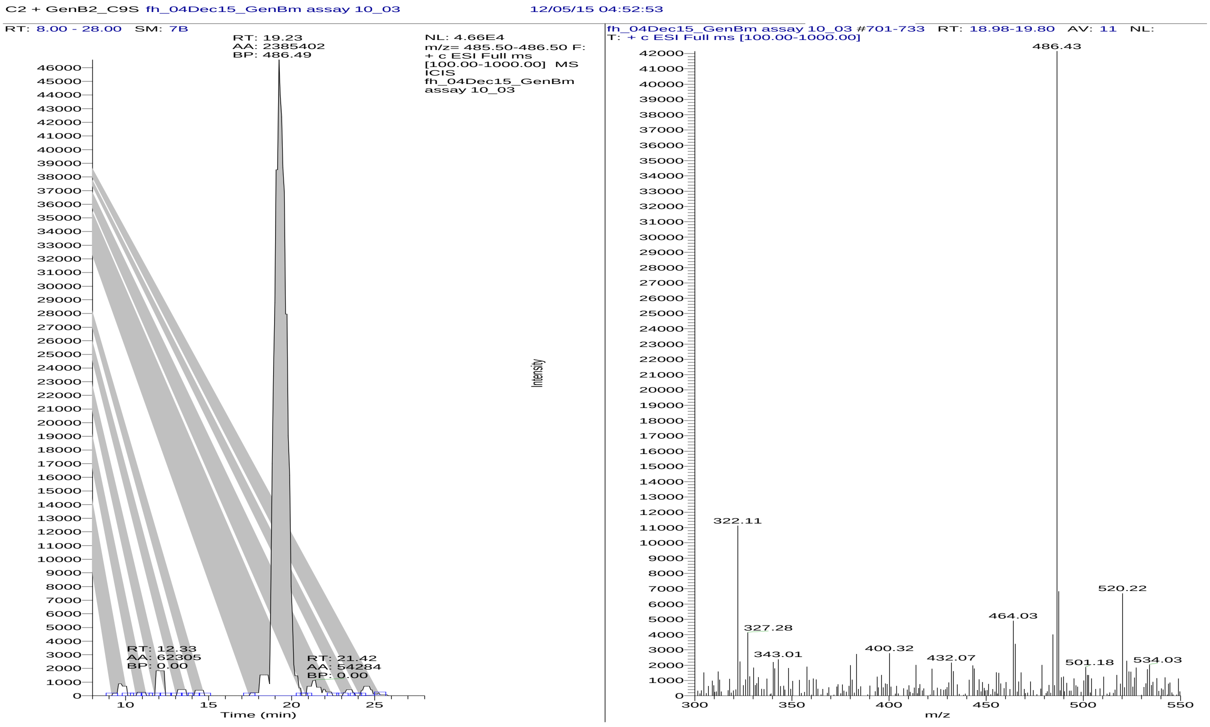


## A4. Gentamicin C2 + GenB2_C9A

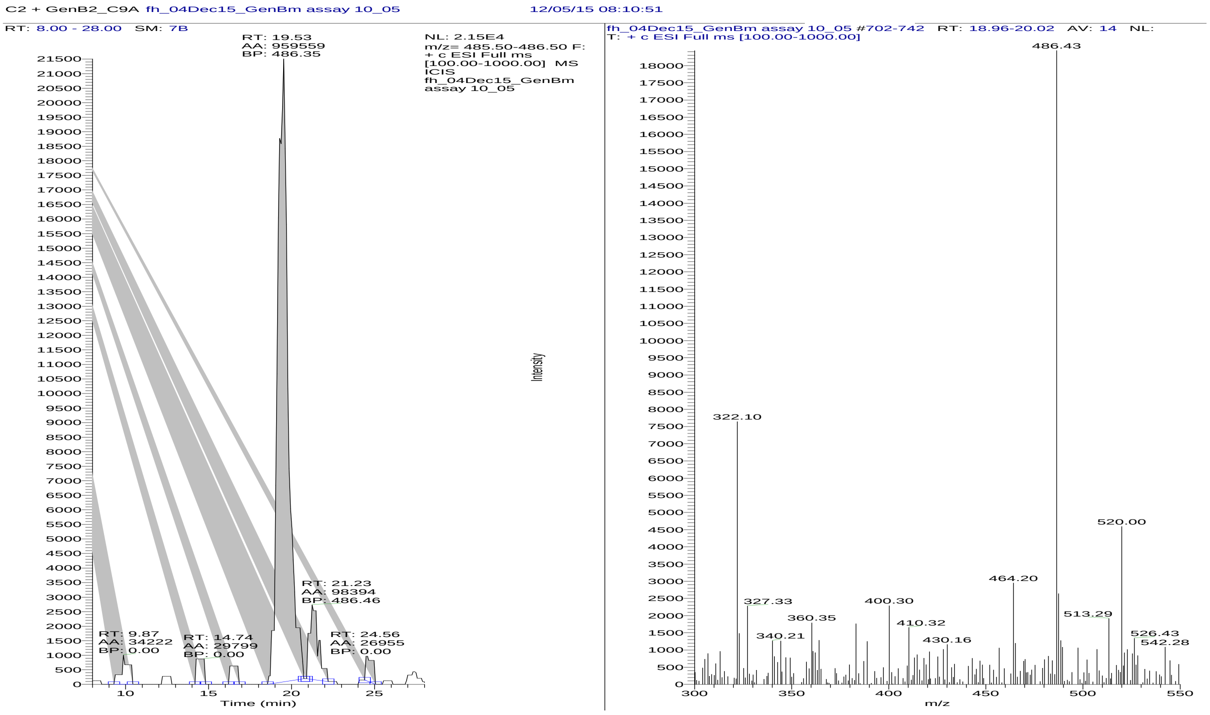

## A5. Gentamicin C2 + GenB2_C9V

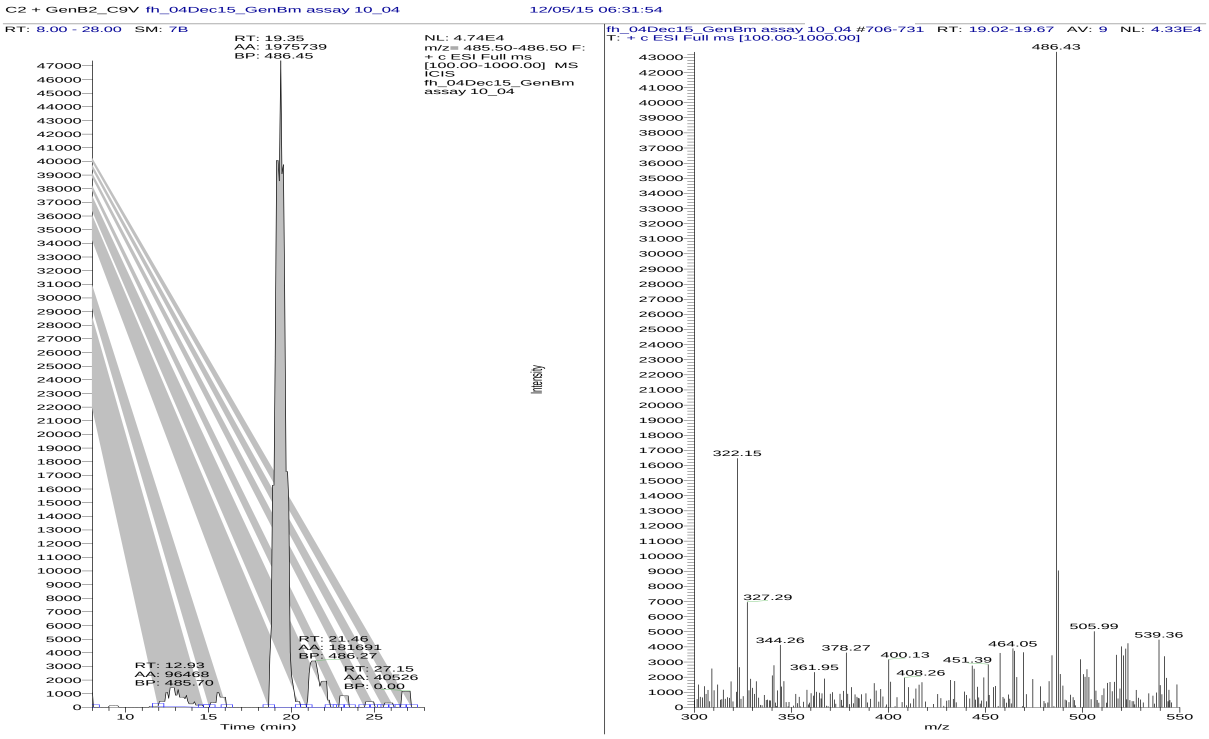


# B. Assays of Gentamicin C2a with GenB2_WT, _C9S, _C9A, _C9V

## B1. Gentamicin C2a without enzyme

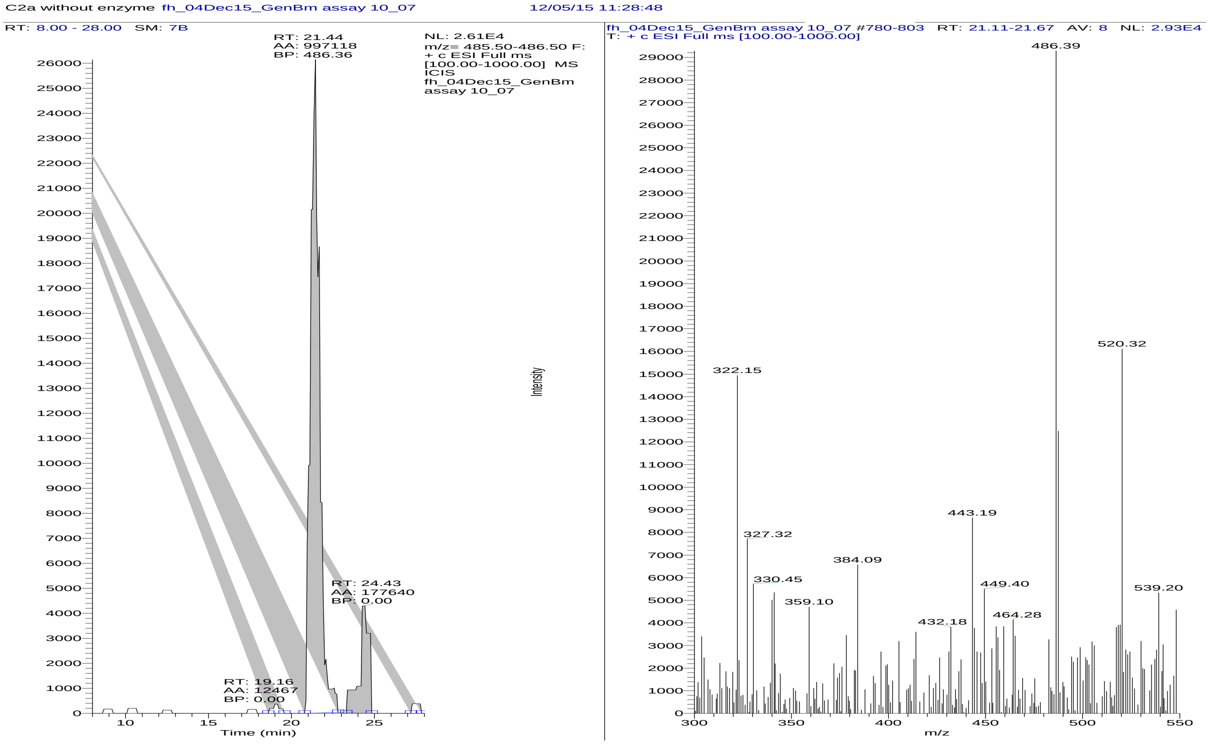

## B2. Gentamicin C2a + GenB2_WT

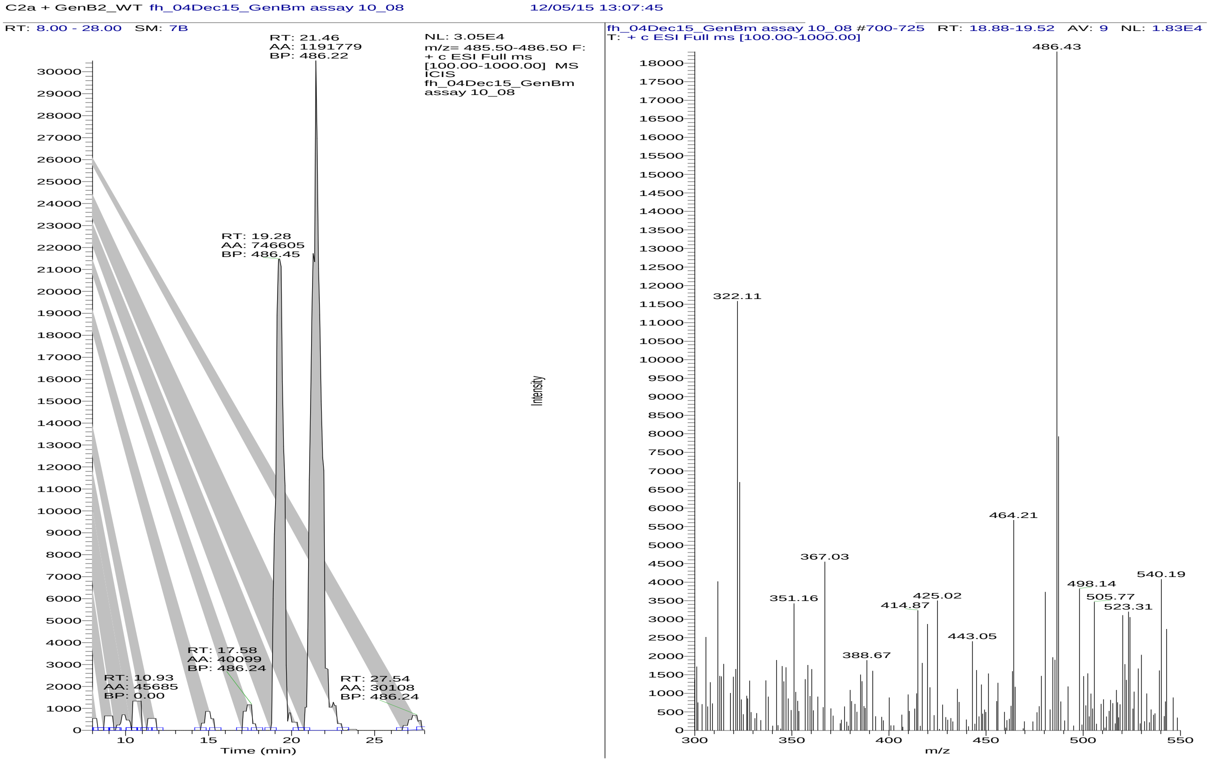


## B3. Gentamicin C2a + GenB2_C9S

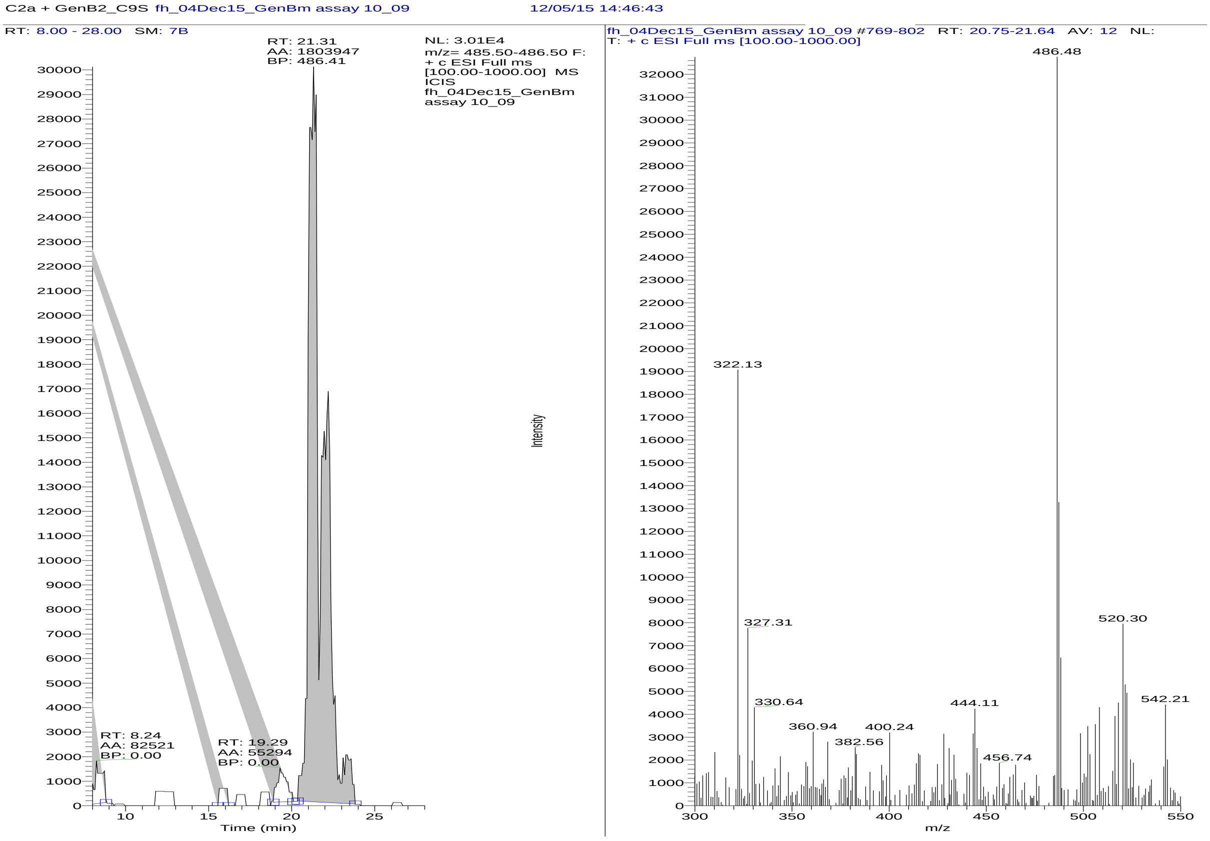

## B4. Gentamicin C2a + GenB2_C9A

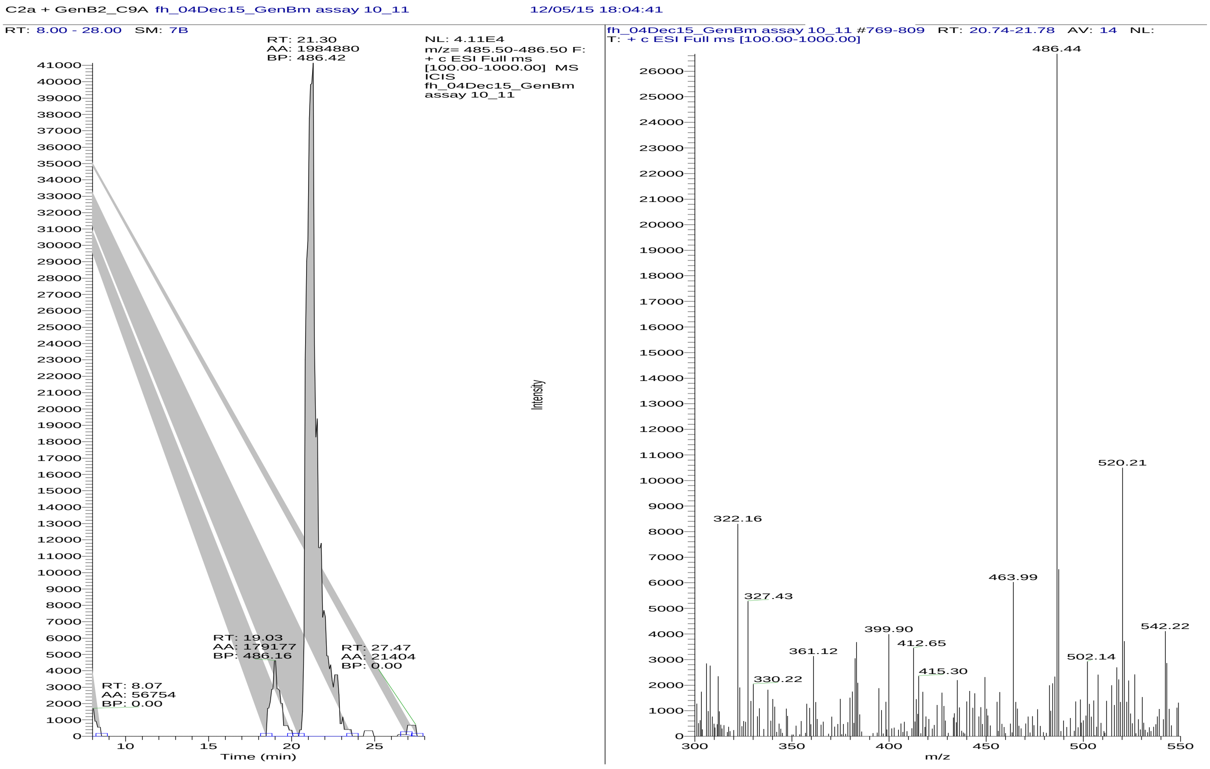


## B5. Gentamicin C2a + GenB2_C9V

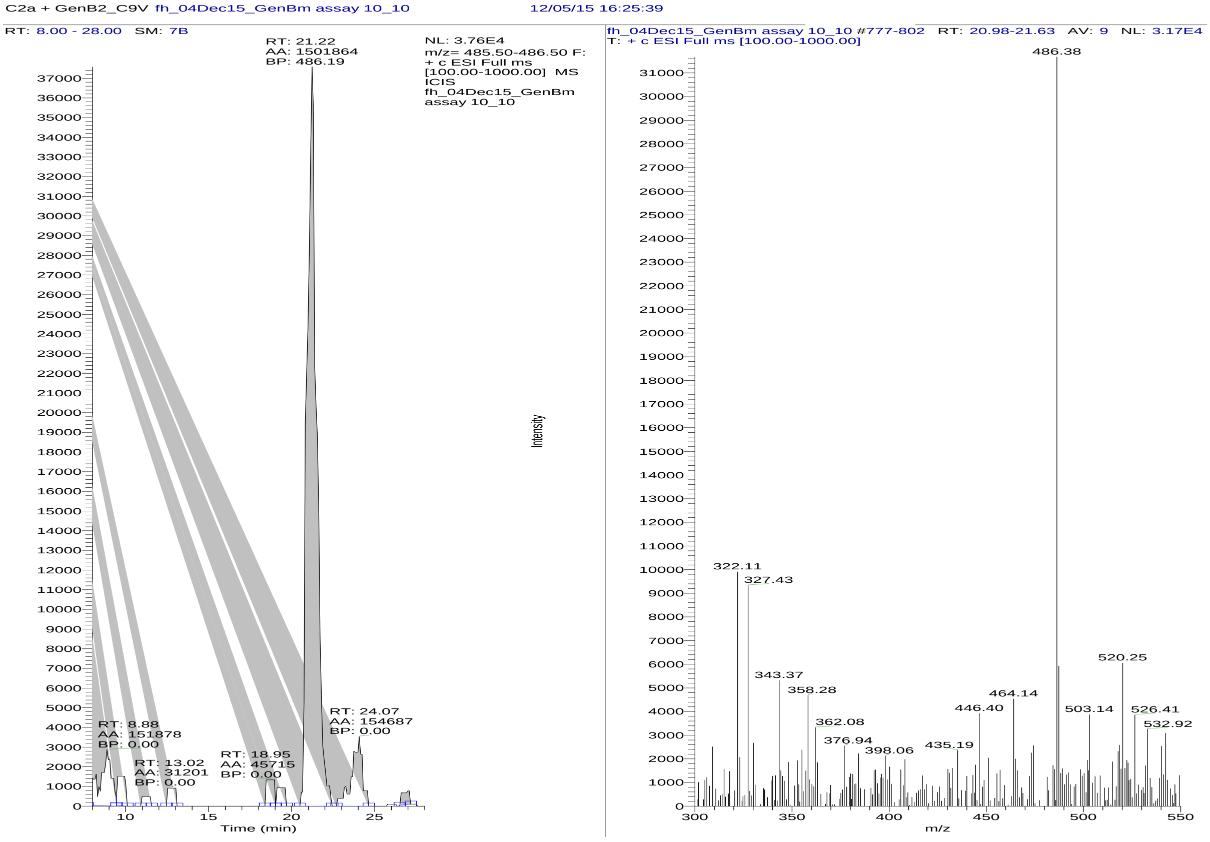

## C. Assays of Gentamicin C2 with GenB2_WT, _F43R, _Y124F, _K227A

### C1. Gentamicin C2 without enzyme

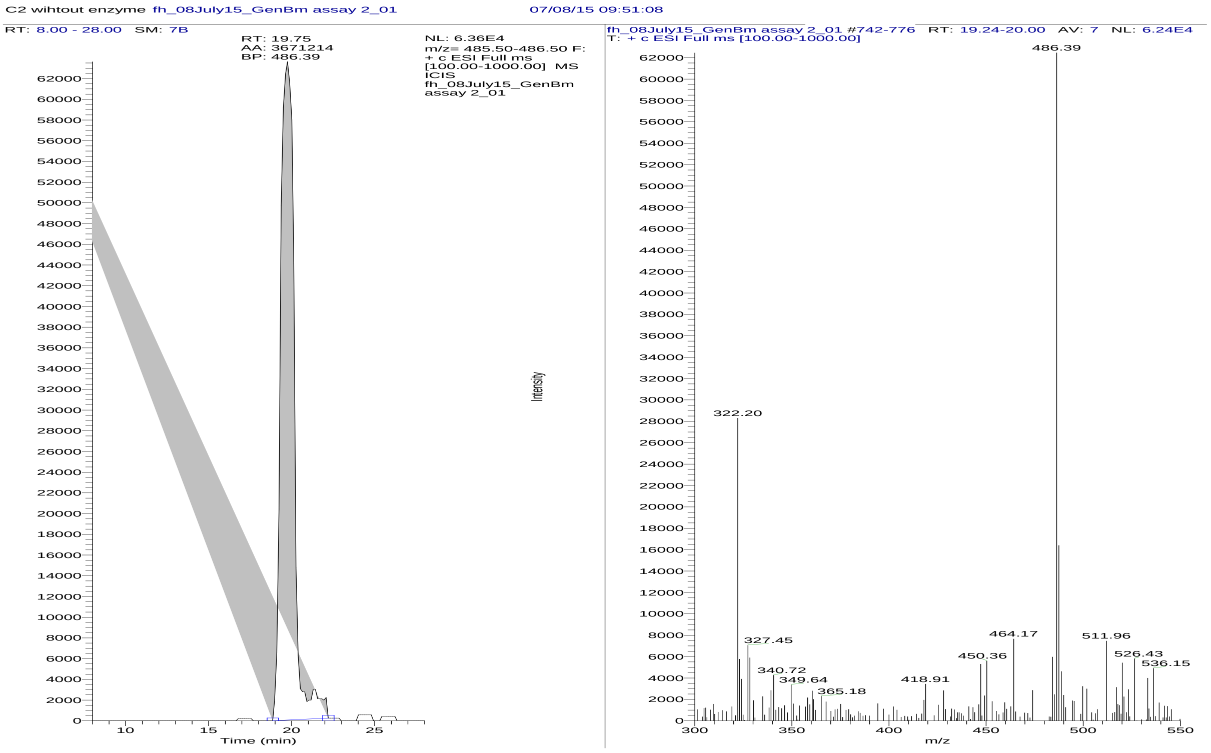


### C2. Gentamicin C2 + GenB2_WT

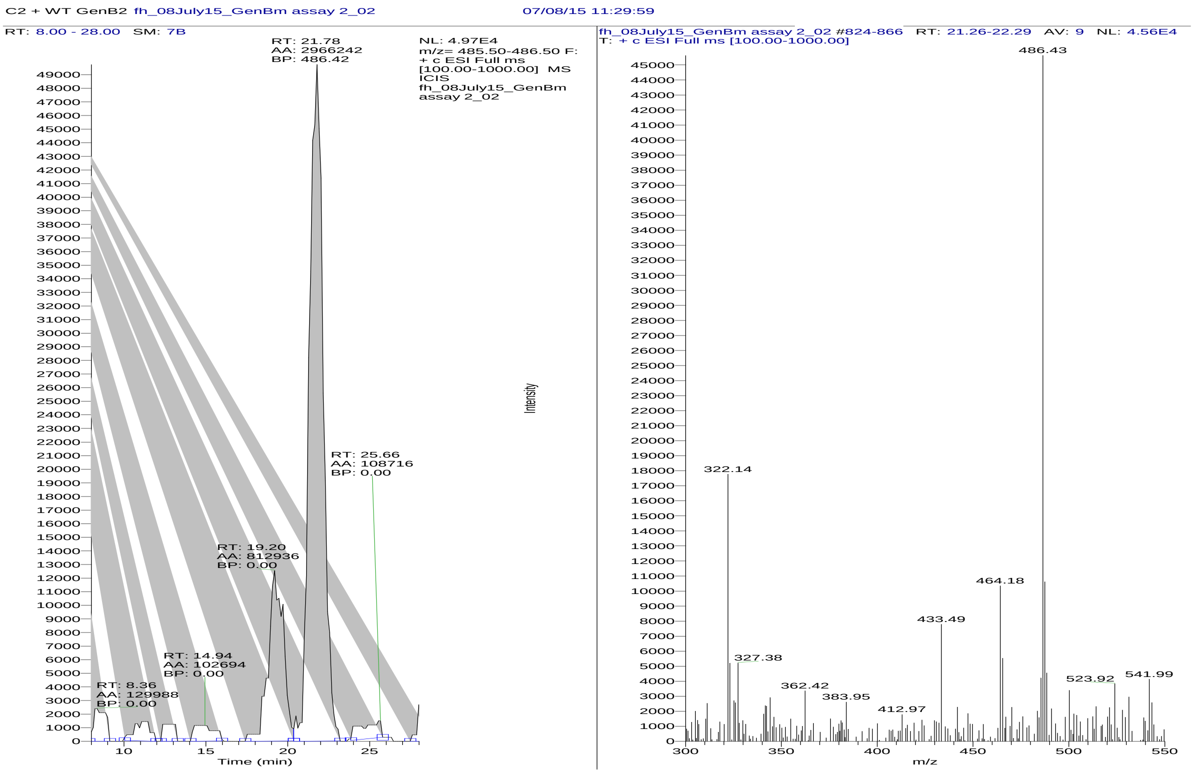

C3. Gentamicin C2 + GenB2_F43R

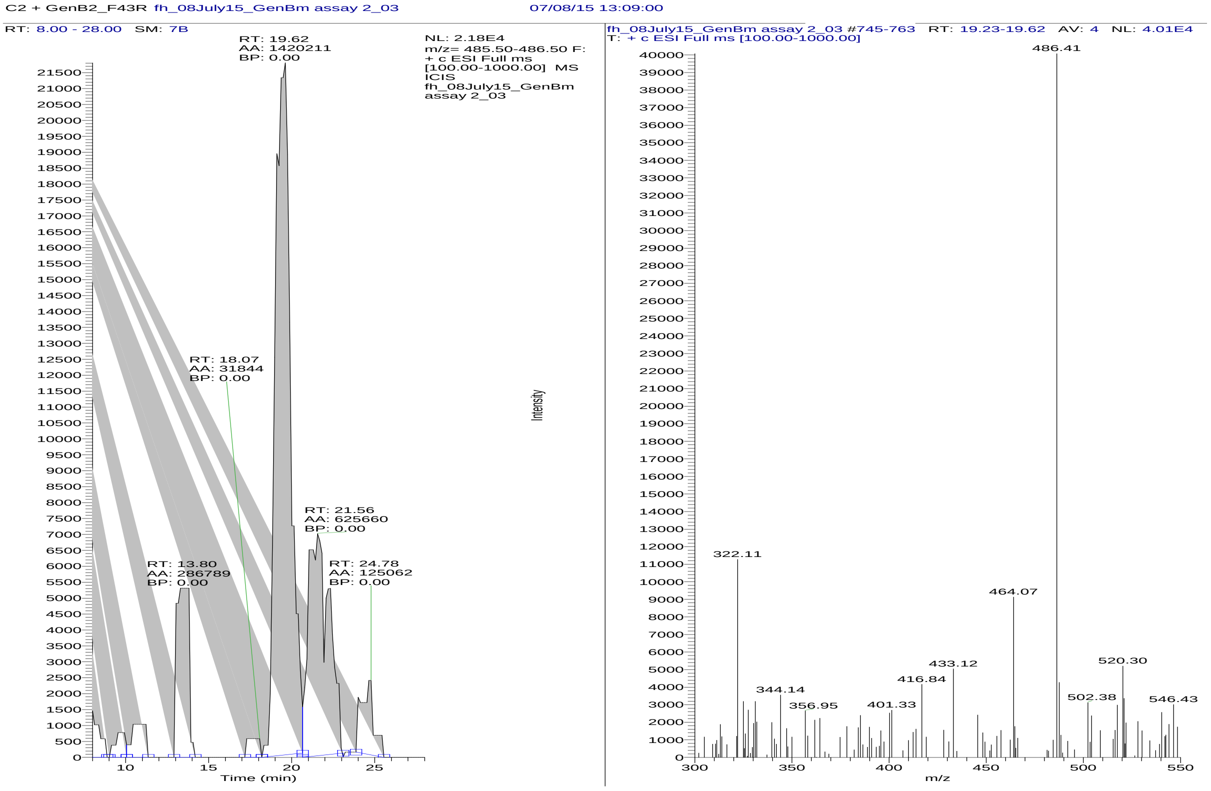


C4. Gentamicin C2 + GenB2_Y124F

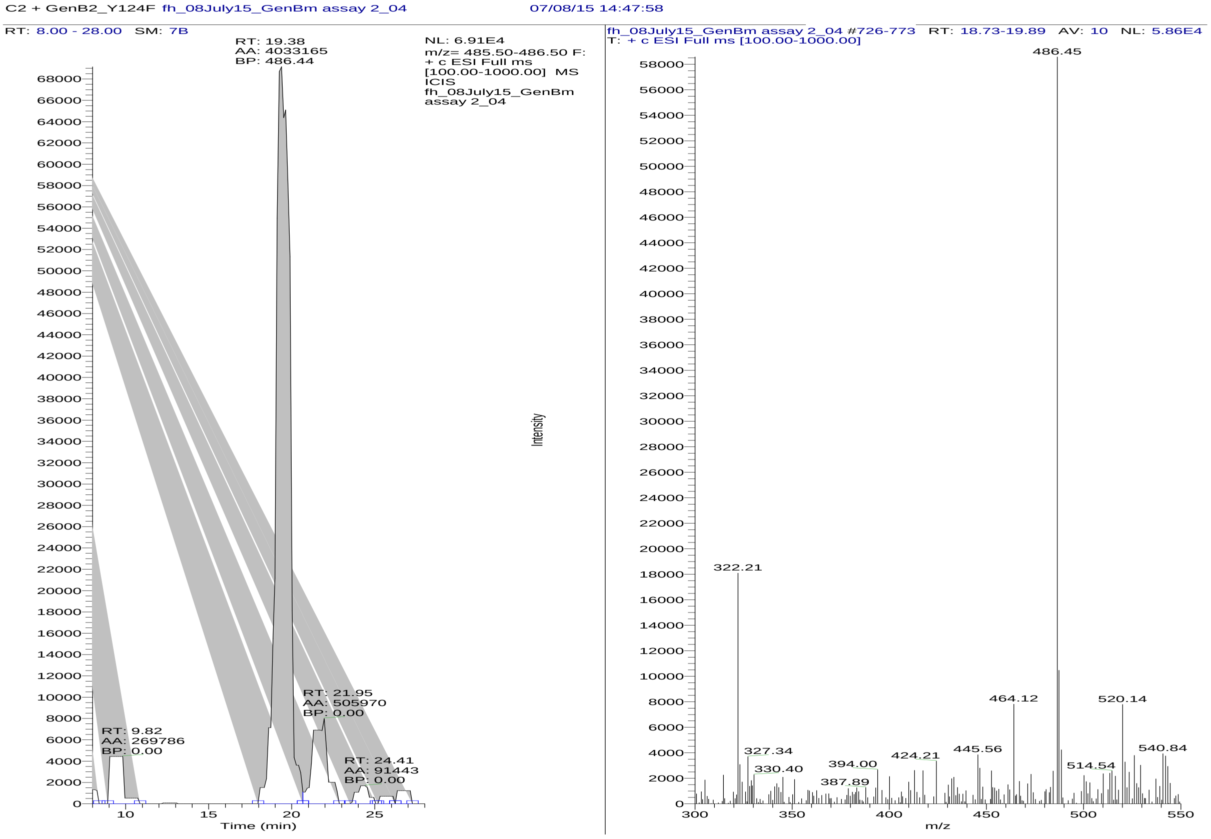

C5. Gentamicin C2 + GenB2_K227A

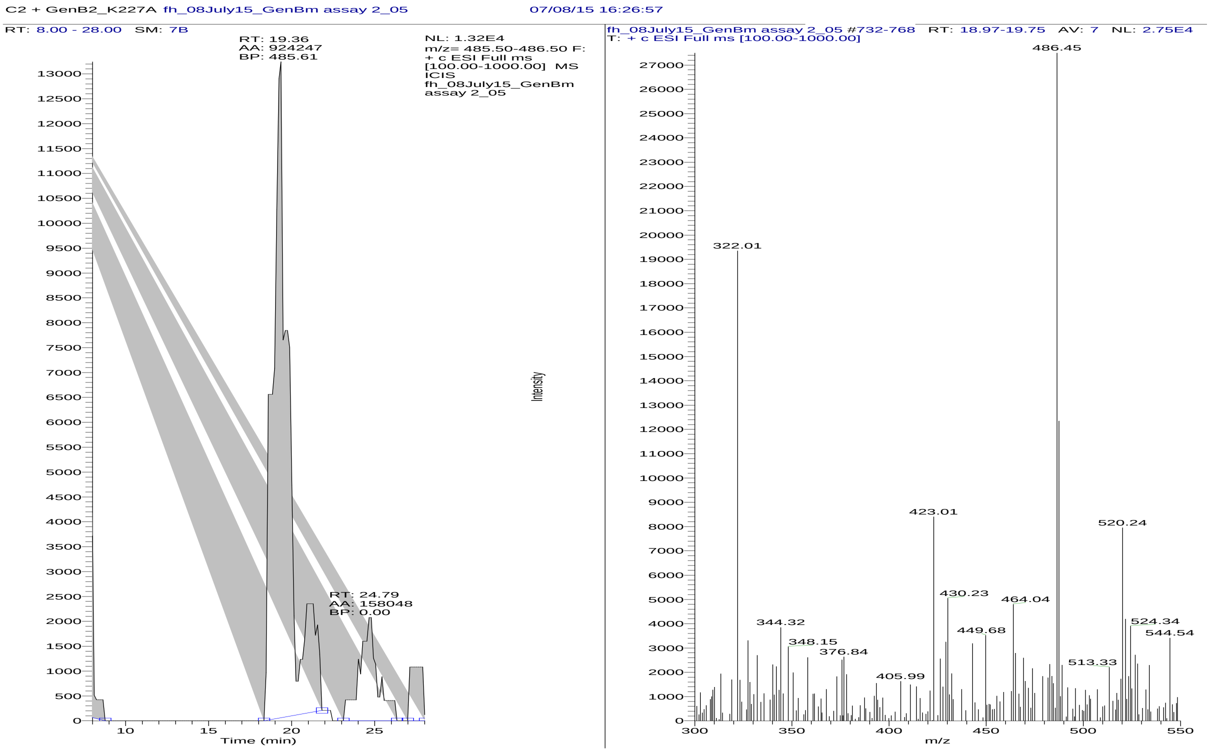


## D. Assays of Gentamicin C2a with GenB2_WT, _F43R, _Y124F, _K227A)

D1. Gentamicin C2a without enzyme

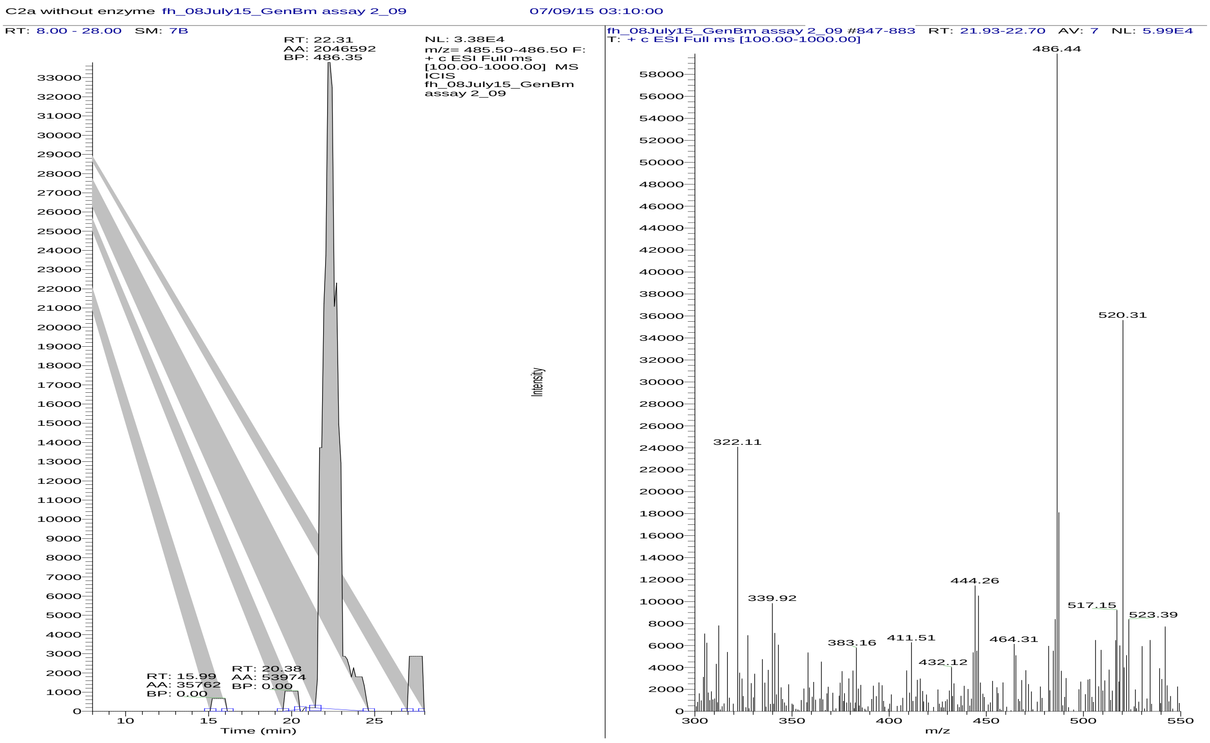

## D2. Gentamicin C2a + GenB2_WT

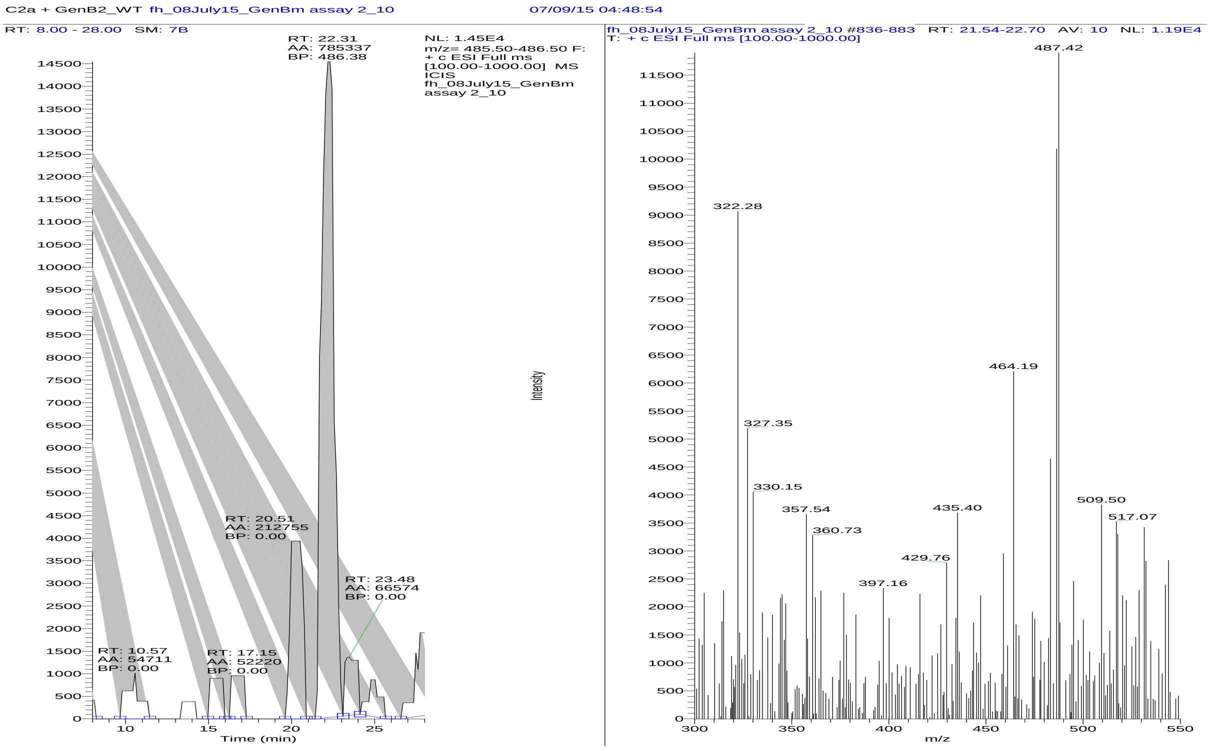


## D3. Gentamicin C2a + GenB2_F43R

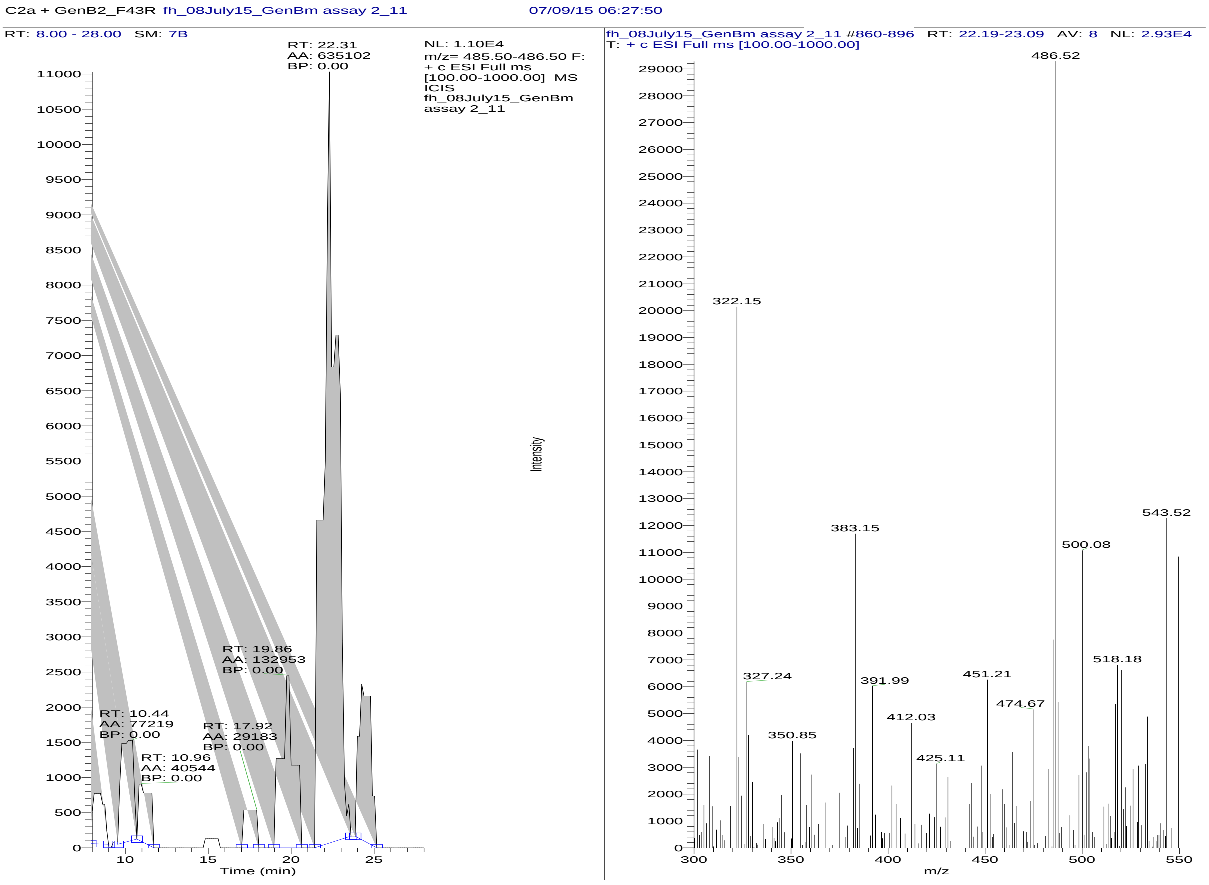

## D4. Gentamicin C2a + GenB2_Y124F

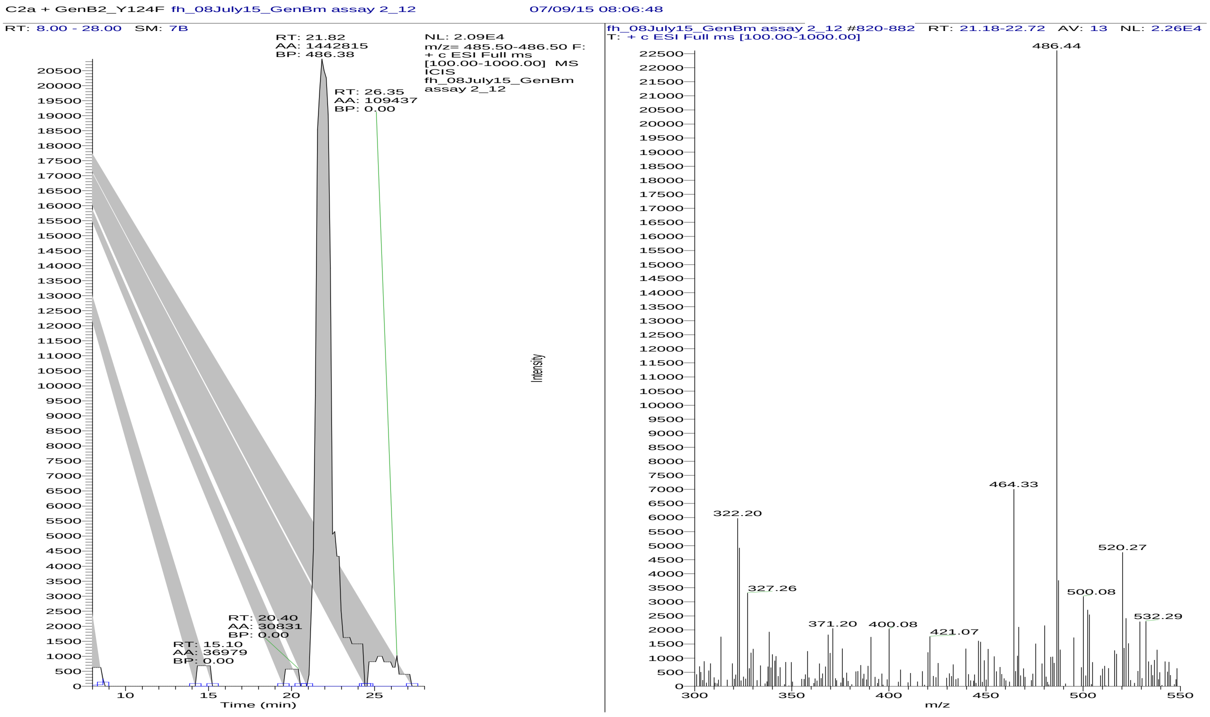


## D5. Gentamicin C2a + GenB2_K227A

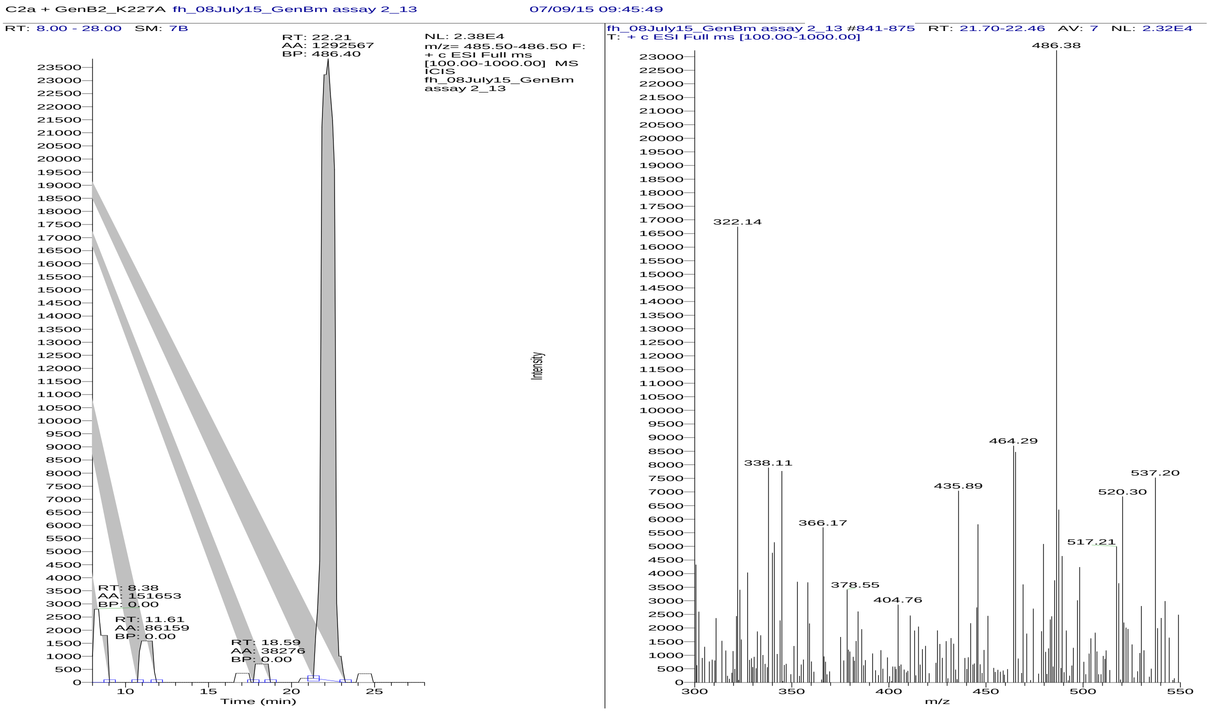

Supplement: Supplementary file 1 — cb4c00334_si_001.pdf [file cb4c00334_si_001.pdf]
